# Nonenzymatic lysine d-lactylation induced by glyoxalase II substrate SLG dampens inflammatory immune responses

**DOI:** 10.1038/s41422-024-01060-w

**Published:** 2025-01-06

**Authors:** Qihang Zhao, Qiang Wang, Qinghua Yao, Zhengdong Yang, Wenfang Li, Xiaojie Cheng, Yingling Wen, Rong Chen, Junfang Xu, Xuanying Wang, Dexiang Qin, Shuyang Zhu, Liujie He, Nan Li, Yanfeng Wu, Yizhi Yu, Xuetao Cao, Pin Wang

**Affiliations:** 1 National Key Laboratory of Immunity & Inflammation, Second Military Medical University, Shanghai, China; 2 Department of Urology, People’s Hospital, Peking University, Beijing, China; 3 The Second Affiliated Hospital of Zhejiang Chinese Medical University, Xinhua Hospital of Zhejiang Province, Zhejiang, China; 4 Department of Emergency and Intensive Care Unit, Changzheng Hospital, Second Military Medical University, Shanghai, China; 5 Clinical and Translational Research Center, Shanghai Pulmonary Hospital, Tongji University School of Medicine, Shanghai, China; 6 Department of Immunology, Institute of Basic Medical Sciences, Chinese Academy of Medical Sciences, Beijing, China; 7 Frontier Research Center for Cell Response, Institute of Immunology, College of Life Sciences, Nankai University, Tianjin, China; 8 National Key Laboratory of Immunity and Inflammation, Suzhou Institute of Systems Medicine, Chinese Academy of Medical Sciences & Peking Union Medical College, Suzhou Jiangsu, China

**Keywords:** Innate immunity, Post-translational modifications, Post-translational modifications

## Abstract

Immunometabolism is critical in the regulation of immunity and inflammation; however, the mechanism of preventing aberrant activation-induced immunopathology remains largely unclear. Here, we report that glyoxalase II (GLO2) in the glycolysis branching pathway is specifically downregulated by NF-κB signaling during innate immune activation via tristetraprolin (TTP)-mediated mRNA decay. As a result, its substrate *S*-D-lactoylglutathione (SLG) accumulates in the cytosol and directly induces d-lactyllysine modification of proteins. This nonenzymatic lactylation by SLG is greatly facilitated by a nearby cysteine residue, as it initially reacts with SLG to form a reversible *S*-lactylated thiol intermediate, followed by *SN*-transfer of the lactyl moiety to a proximal lysine. Lactylome profiling identifies 2255 lactylation sites mostly in cytosolic proteins of activated macrophages, and global protein structure analysis suggests that proximity to a cysteine residue determines the susceptibility of lysine to SLG-mediated d-lactylation. Furthermore, lactylation is preferentially enriched in proteins involved in immune activation and inflammatory pathways, and d-lactylation at lysine 310 (K310) of RelA attenuates inflammatory signaling and NF-κB transcriptional activity to restore immune homeostasis. Accordingly, TTP-binding site mutation or overexpression of GLO2 in vivo blocks this feedback lactylation in innate immune cells and promotes inflammation, whereas genetic deficiency or pharmacological inhibition of GLO2 restricts immune activation and attenuates inflammatory immunopathology both in vitro and in vivo. Importantly, dysregulation of the GLO2/SLG/d-lactylation regulatory axis is closely associated with human inflammatory phenotypes. Overall, our findings uncover an immunometabolic feedback loop of SLG-induced nonenzymatic d-lactylation and implicate GLO2 as a promising target for combating clinical inflammatory disorders.

## Introduction

A properly mounted immune response is crucial for recognizing and eliminating danger arising from sterile and microbial injuries. However, the ‘inflammatory fire’ triggered by the immune response must be tightly controlled to prevent it from spreading and causing irreversible damage.^1,2^ Accordingly, acute inflammation is always transiently generated and self-limited under normal conditions to restore homeostasis. In contrast, unbridled inflammation can lead to the development of sepsis, autoimmunity, and degenerative diseases. Although intracellular self-limiting mechanisms have been revealed at the level of transcription, epigenetics, RNA degradation, and protein modifications,^3–5^ a well understanding of the role of immunometabolism in this process is still lacking.^6^ The glyoxalase system is an enzymatic network in mammals that detoxifies reactive metabolites, such as methylglyoxal (MGO), which is the most common byproduct of glycolysis.^7^ However, there is limited understanding of the impact of the glyoxalase system, particularly glyoxalase II (GLO2) and its substrate *S*-D-lactoylglutathione (SLG), on immune activation and inflammation regulation.

Mounting evidence suggests that metabolites or intermediates that accumulate in immune cells can modulate immune responses by directly interacting with or modifying intracellular proteins, which are called non-canonical (“moonlighting”) functions.^8,9^ Thus far, there has been a focus on intermediates in the mitochondria, particularly Krebs cycle metabolites.^10^ Indeed, itaconate^11,12^ and fumarate^13–15^ have recently emerged as important regulators of inflammation and immunity in multiple contexts. Itaconate and fumarate can directly modify their target proteins after moving out from the mitochondria. However, it is largely unknown whether there are any other metabolites or metabolic intermediates in the cytosol that could react with immune mediators in situ, and thus regulate inflammatory signaling and immune activation.

Lactylation (lacK) modification was first identified on histone lysine residues as l-lactylation in macrophages to regulate gene expression during activation.^16,17^ Recently lactylation on nonhistone proteins was functionally characterized. It has been shown that lactylation at lysine 673 of the homologous recombination protein MRE11 exerts a key function in regulating its DNA-binding ability and subsequent DNA end resection.^18^ In cancer cells, lactylation of p53 in the DNA-binding domain hinders the liquid–liquid phase separation, DNA binding, and transcriptional activation of p53.^19^ Hypoxia in myoblasts induces the lactylation of mitochondrial enzymes PDHA1 and CPT2 to attenuate their activity and inhibit oxidative phosphorylation.^20^ In these studies, l-lactylation is believed to be induced by l-lactate through an enzyme-dependent manner and several lactyltransferases have been identified, such as histone acetyltransferase P300 and CBP, and alanyl-tRNA synthetase AARS1/2.^18–21^ Here, our research uncovered a unique d-lactylation process induced by SLG in activated innate immune cells. This nonenzymatic reaction is characterized by the initial lactylation of a nearby cysteine residue, followed by *SN*-transfer of the lactyl moiety to the proximal lysine. SLG accumulates due to the dramatic downregulation of GLO2 through NF-κB-dependent TTP-mediated mRNA decay in inflammation. This lactylation on immune mediators acts as a brake on immune activation and inflammation. Thus, SLG-induced d-lactylation is part of an immunometabolic autoregulatory loop that constrains the inflammatory response, and dysregulation of the GLO2/SLG/d-lactylation regulatory axis is closely related to severe inflammatory phenotypes and associated with poor prognosis.

## Results

### GLO2 levels decrease in activated immune cells and are significantly associated with severe inflammatory diseases

**Fig. 1:**
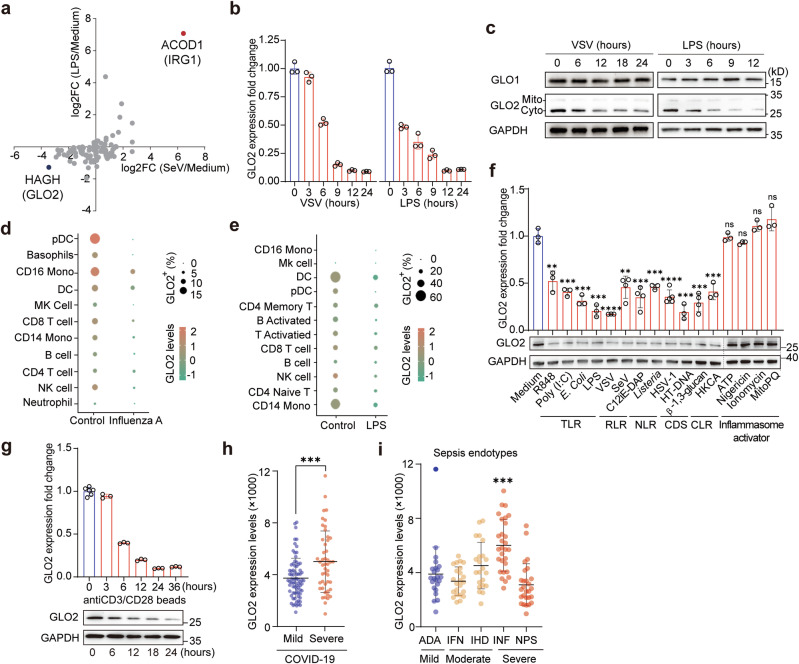
GLO2 is downregulated after immune activation in immune cells. **a** RNA-seq data profiling of metabolic enzymes in mouse macrophages stimulated by Sendai virus (SeV) (12 h, MOI = 1) or LPS (6 h, 100 ng/mL). The untreated control group (Medium) is set to a value of 1. **b**, **c** Q-PCR (**b**) and Immunoblotting (**c**) analysis of indicated gene expression in BMDMs infected by VSV (MOI = 1) or LPS for indicated time points. Unless noted otherwise, all results in this and other figures were representative of at least three independent experiments. Q-PCR data are normalized to hypoxanthine-guanine phosphoribosyltransferase (HPRT) levels, and the medium group is set to a value of 1. Unless noted otherwise, the error bars in this and all other panels denote SD. **d**, **e** scRNA-seq analysis of GLO2 levels in different immune cells from human PBMCs infected by influenza A virus (GSE243629) (**d**) or stimulated by LPS (GSE226488) (**e**). **f** Q-PCR and immunoblot analysis of indicated gene expression in BMDMs stimulated as indicated. HKCA heat-killed preparation of *Candida albicans*, MitoPQ MitoParaquat. **g** Q-PCR and immunoblot analysis of indicated gene expression in CD3^+^ T cells separated from human PBMCs and then stimulated by anti-CD3/CD28 beads for indicated time points. **h** GLO2 levels in the expression profiling data of leukocytes from 124 patients with mild or severe COVID-19 (GSE221234). **i** GLO2 levels in leukocytes from patients with different sepsis endotypes in COVID-19. ADA Adaptive, IFN Interferon, IHD Innate-Host-Defense, INF Inflammatory, NPS Neutrophilic-Suppressive.

To gain a better understanding of how immunometabolism and immune activation are coordinated, we first conducted a comprehensive investigation on the expression profiles of metabolic enzymes in viral- or bacterial-activated macrophages. As previously reported, aconitate decarboxylase 1 (ACOD1) was most significantly increased as a signature of typical innate immune activation.^14,22^ On the other hand, the most remarkable decrease was revealed to be glyoxalase enzyme GLO2 (Fig. 1a; Supplementary information, Fig. S1a, b), followed by fumarate hydratase (FH1)^14^ and fatty acid synthase (FASN)^23^ whose roles in innate immunity had already been well reported.

GLO2 is encoded by the hydroxyacylglutathione hydrolase (*HAGH*) gene, which is highly conserved in both humans and mice and consists of two isoforms, residing in the cytosol and the mitochondria, respectively.^24^ We confirmed that GLO2 was significantly downregulated at the mRNA and protein levels hours after both viral and bacterial stimulation in macrophages (Fig. 1b, c). The prominent decrease was largely due to the decline of its cytosolic isoform, which comprised over 90% of the total GLO2 in immune cells (Fig. 1b, c; Supplementary information, Fig. S1c–e). However, the expression of the other glyoxalase enzyme, glyoxalase I (GLO1), was barely changed after immune activation (Fig. 1c; Supplementary information, Fig. S1f).

To examine the expression level of GLO2 in different immunocytes, we investigated single-cell RNA sequencing (scRNA-seq) data of peripheral blood mononuclear cells (PBMCs) from healthy individuals and individuals with Influenza A virus (IAV) infection and scRNA-seq data of PBMCs with or without lipopolysaccharide (LPS) stimulation. We found that GLO2 levels were consistently downregulated upon viral or bacterial stimulation in both innate and adaptive immune cells, with mono/macrophages and dendritic cells showing the most significant decline (Fig. 1d, e; Supplementary information, Fig. S1g, h) which was also confirmed by quantitative real-time PCR (Q-PCR) (Supplementary information, Fig. S1i).

Immune cells encounter various stimuli, stresses, or different antigens under physiological or pathological conditions. Therefore, we first examined the effect of various “danger signals” on GLO2 in innate immune cells. We found that pattern recognition receptor (PRR) signaling could induce an obvious downregulation of GLO2 in macrophages, while LPS and vesicular stomatitis virus (VSV) treatment exhibited the most marked decrease (Fig. 1f). In contrast, stresses, inflammasome activators or signal 2 activators, including ATP, Nigericin, and ROS, did not affect GLO2 levels solely. Then we investigated the impact of antigen signaling from T-cell receptor (TCR) on GLO2 in human T cells. We observed that T-cell activation induced by anti-CD3/CD28 beads resulted in GLO2 downregulation at both mRNA and protein levels (Fig. 1g). These data indicate that GLO2 downregulation is a common phenomenon following immune activation in both innate and adaptive immune cells.

Next, we tested the physiological and pathological relevance of this finding by analyzing GLO2 levels in mouse and human inflammatory diseases. Intriguingly, GLO2 levels were dramatically decreased in the spleen and lung of K18-hACE2 mice intranasally infected with severe acute respiratory syndrome coronavirus 2 (SARS‑CoV‑2) (Supplementary information, Fig. S1j), indicating that GLO2 downregulation may be involved in the regulation of immune responses. Then, we analyzed the expression profiling data of 124 COVID-19 patients and found that severe COVID-19 patients showed higher GLO2 levels in their leukocytes than patients with mild symptoms (Fig. 1h). This suggests that elevated GLO2 levels in immune cells might contribute to excessive immune activation and worsening inflammation. We, therefore, examined the expression data of human PBMCs with sepsis, an immune-dysregulation disease with heterogeneous degrees of immunosuppressive or overactivation in different endotypes.^25^ We found that the endotype with immune overactivation (inflammatory, INF) had the highest GLO2 levels in their peripheral leukocytes, and, on the contrary, the severe immunosuppressive endotype (Neutrophilic-Suppressive, NPS) had the lowest GLO2 levels (Fig. 1i), suggesting that GLO2 level is closely associated with inflammatory level. These data demonstrate that glyoxalase enzyme GLO2 may play a role in immune homeostasis and a well-controlled immune response.

### NF-κB-induced TTP binds to the ARE of *GLO2* mRNA to mediate its decay

**Fig. 2:**
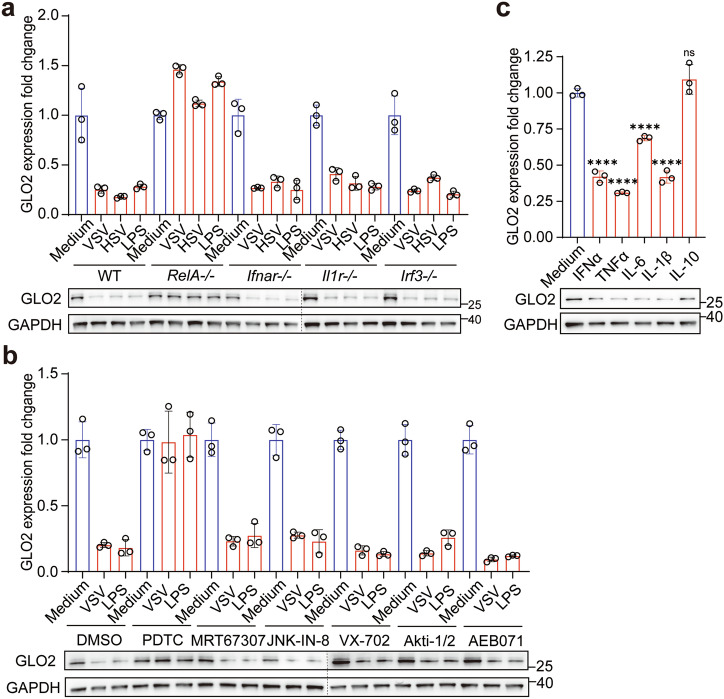
Downregulation of GLO2 depends on the NF-κB signaling pathway. **a** Q-PCR and immunoblot analysis of indicated gene expression in WT or gene-knockout BMDMs stimulated as indicated. **b** Q-PCR and immunoblot analysis of indicated gene expression in BMDMs pretreated with indicated inhibitors for 1 h and then stimulated by VSV or LPS. **c** Q-PCR and immunoblot analysis of indicated gene expression in BMDMs stimulated by recombinant cytokines.

Next, we further investigated the molecular mechanism that mediates the downregulation of GLO2 in immune cells. First, we used mouse strains with a deficiency in different pathways and found that PRR stimuli did not reduce GLO2 levels in *Rela*^*−/−*^ macrophages (Fig. 2a). In addition, pharmacologic profiling using inhibitors of different pathways revealed that only the nuclear factor-kappa B (NF-κB) inhibitor Ammonium pyrrolidinedithiocarbamate (PDTC) abolished GLO2 downregulation at both mRNA and protein levels (Fig. 2b), indicating that NF-κB signaling is significant in modulating GLO2 level in immune cells. However, in addition to PRR signaling, NF-κB can be activated by members of many immune signaling pathways, including various cytokine receptors, TNF receptor (TNFR) superfamily members, and TCR. We found cytokines that induce NF-κB activation, such as type I interferon, TNFα, IL-6, and IL-1β, all induce GLO2 downregulation in innate immune cells, while the anti-inflammatory cytokine IL-10 does not have this effect (Fig. 2c). These data suggest that NF-κB signaling that induces GLO2 downregulation could be triggered by multiple stimuli during immune activation, including pathogen-associated molecular patterns (PAMP), damage-associated molecular patterns (DAMP), inflammatory cytokines and specific antigens. The combination of these stimuli could result in a greater decrease in the expression of GLO2 (Supplementary information, Fig. S2a, b)

**Fig. 3:**
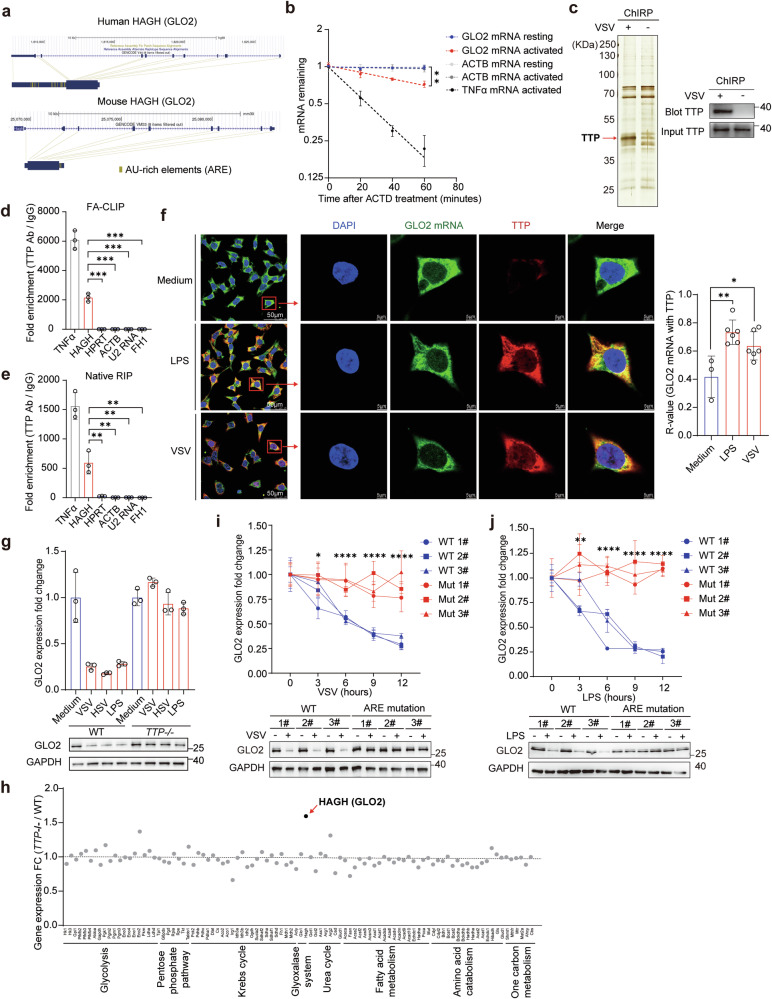
TTP binds to *GLO2* mRNA and mediates its decay. **a** Diagram of AU-rich elements (AREs) in the human and mouse *GLO2* 3′-UTRs. **b** Q-PCR analysis of indicated mRNAs in resting or LPS-activated (6 h, 100 ng/mL) BMDMs treated with ACTD (5 μM) for indicated time points. **c** *GLO2* mRNA along with its binding proteins were retrieved (through ChIRP) in resting or VSV-activated human THP-1-derived macrophages and the specific band was identified by MS or immunoblot with a TTP antibody. **d**, **e** Q-PCR analysis of indicated mRNAs retrieved by TTP antibody or control antibody from BMDMs under crosslinked (**d**) or native (**e**) conditions. **f** RNA FISH of *GLO2* mRNA and immunofluorescence assay of TTP protein in human THP-1-derived macrophages stimulated with LPS, VSV or not (medium). Scale bars, 5 μm. The co-localization was analyzed by ImageJ. **g** Q-PCR and immunoblot analysis of GLO2 expression in WT and *TTP*^*−/−*^ BMDMs stimulated as indicated. **h** RNA-seq data profiling of metabolic enzymes in WT and *TTP*^*−/−*^ BMDMs stimulated by LPS for 6 h (GSE63468). **i**, **j** Q-PCR and immunoblot analysis of GLO2 expression in WT or GLO2 ARE-sites mutant RAW 264.7 cell lines stimulated by VSV (**i**) or LPS (**j**) for indicated time points.

Next, we analyzed the mRNA sequence of GLO2 and found that both the human and mouse 3′UTRs contain several AU-rich elements (AREs) (Fig. 3a). ARE-binding proteins and ARE-directed mRNA degradation play an important role in the rapid resolution of increased inflammatory cytokines, such as TNFα and IL-6.^26,27^ Therefore, we first performed mRNA stability assays using Actinomycin D (ACTD) to inhibit mRNA synthesis and found that *GLO2* mRNA stability was indeed significantly decreased after macrophage activation (Fig. 3b). On the contrary, the protein degradation of GLO2 after translation inhibition induced by cycloheximide (CHX) was not affected by innate immune activation (Supplementary information, Fig. S2c). To identify the candidate proteins that bind to *GLO2* mRNA during immune activation, we then performed endogenous RNA pulldown assay (ChIRP) followed by protein mass spectrometry (MS) identification (Fig. 3c left). Tristetraprolin (TTP), an ARE-binding protein that mediates mRNA decay, was found to be associated with the *GLO2* mRNA in activated macrophages (Fig. 3c right; Supplementary information, Fig. S2d–f). Consistently, bioinformatic analysis showed that the 3′ UTR region of GLO2 contains multiple TTP-binding sites (Supplementary information, Fig. S2g, h), among which two top-scored AREs are highly conserved in humans and mice. To further validate their interaction, crosslinked RNA immunoprecipitation (FA-CLIP) and native RNA immunoprecipitation (RIP) assays were both performed using a TTP-specific antibody, and *GLO2* mRNA, as well as *TNFα* mRNA, was found to bind to TTP protein (Fig. 3d, e; Supplementary information, Fig. S2i–l). Furthermore, fluorescence imaging shows the co-localization of *GLO2* mRNA and TTP protein in the cytoplasm of LPS- or VSV-activated macrophages (Fig. 3f).

TTP was previously reported to be activated by NF-κB in LPS-stimulated macrophages.^28^ We confirmed that TTP was upregulated by different PRR stimuli in immune cells (Supplementary information, Fig. S3a), and its expression was mediated by NF-κB signaling (Supplementary information, Fig. S3b–d). Dynamic detection in macrophages revealed a negative correlation between TTP and GLO2 levels during viral or bacterial stimulation (Supplementary information, Fig. S3e, f).

We hypothesized that various stimuli causing NF-κB activation lead to the induction of TTP expression, which then binds to the ARE of *GLO2* mRNA and facilitates its decay. To test this, we conducted experiments on macrophages with TTP deficiency or siRNA-mediated knockdown. The results showed that the decrease in GLO2 levels induced by immune activation was significantly reduced in the absence of TTP (Fig. 3g; Supplementary information, Fig. S3g). Furthermore, metabolic enzyme profiling in LPS-treated TTP-deficient macrophages revealed that GLO2 was the most significantly upregulated (Fig. 3h). To make our findings more convincing, we created RAW 264.7 macrophage cell lines with an ARE mutation on the *Hagh* 3′UTR to specifically prevent TTP binding to *GLO2* mRNA. Our results showed that the mutation in the two highly conserved TTP-binding sites successfully prevented the downregulation of GLO2 after innate immune activation (Fig. 3i, j). These data confirm that the binding of TTP to these two AREs of *GLO2* mRNA is responsible for the decrease in GLO2 in immune cells following activation. However, *FH1* mRNA, which was also decreased in activated macrophages, was not influenced by TTP deficiency and was not associated with TTP (Fig. 3d, e; Supplementary information, Fig. S3h), indicating that its downregulation is not via TTP-mediated mRNA decay.

### Reduced GLO2 leads to the accumulation of SLG in activated immune cells

**Fig. 4:**
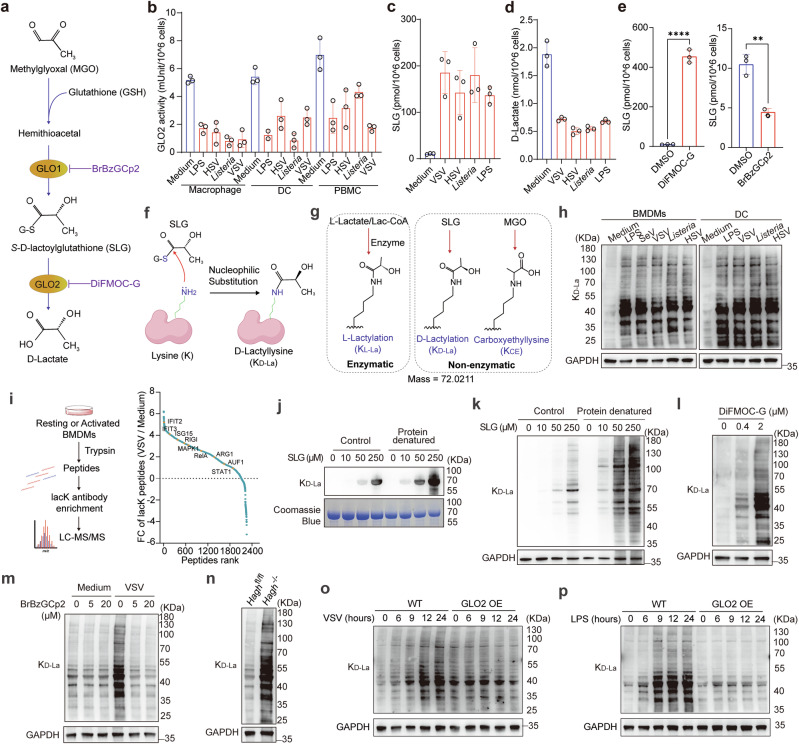
SLG accumulates after GLO2 downregulation and directly induces nonenzymatic d-lactylation. **a** Diagram of GSH-dependent glyoxalase pathway and the corresponding pharmacological inhibitors. **b** Enzymatic activity detection of GLO2 in innate immune cells stimulated as indicated. **c** Targeted LC–MS quantification of SLG in BMDMs stimulated as indicated. **d** Relative quantification of D-Lactate in BMDMs stimulated as indicated. **e** LC–MS quantification of SLG in BMDMs treated with DiFMOC-G (0.4 μM) or BrBzGCp2 (5 μM) for 24 h. **f** Schematic outlines of nucleophilic substitution reaction between SLG and a lysine residue. **g** Schematic diagram of the three isomer modifications and their different metabolic derivation: K_L-La_, K_D-La_, and K_CE_. **h** Immunoblot of K_D-La_ levels in innate immune cells stimulated as indicated for 12 h. **i** Diagram of antibody-enriched lacK peptide identification by LC–MS/MS in BMDMs (left) and fold change of the identified lacK peptides by VSV activation. *n* = 3 (right). **j**, **k** Immunoblot of K_D-La_ levels in BSA (**j**) or macrophage lysate (**k**) denatured by boiling or not, then co-incubated with indicated concentration of SLG for 4 h at 37 °C. **l** Immunoblot of K_D-La_ levels in BMDMs pretreated with indicated concentrations of DiFMOC-G for 24 h. **m** Immunoblot of K_D-La_ levels in BMDMs pretreated with indicated concentrations of BrBzGCP2 then stimulated by VSV or not. **n** Immunoblot detection of K_D-La_ levels in *Hagh*^+/+^ and *Hagh*^*−/−*^ BMDMs. **o**, **p** Immunoblot of K_D-La_ levels in BMDMs from WT and GLO2-overexpression (GLO2 OE) mice stimulated by VSV (**o**) or LPS (**p**) for indicated time points.

The glyoxalase system consists of two enzymes, GLO1 and GLO2. The enzyme GLO1 is the primary enzyme involved in the glutathione (GSH)-dependent detoxification of MGO. It catalyzes the isomerization of an intermediate hemithioacetal formed by MGO and glutathione to the corresponding α-d-hydroxyacid thioester, which is non-toxic SLG. GLO2 plays a supportive role in the glyoxalase system, mainly by hydrolyzing SLG to regenerate GSH and produce d-lactate (Fig. 4a).^29,30^ After immune activation, as its protein level decreases, we observed a dramatic decline in GLO2 hydrolase activity per cell in innate immune cells, while GLO1 enzymatic activity remained largely unchanged (Fig. 4b; Supplementary information, Fig. S4a). Consequently, the GLO2 substrate SLG accumulated significantly in activated macrophages (Fig. 4c), as indicated by targeted metabolite detection. However, SLG levels in the culture medium were barely detected after activation, indicating its low cytomembrane permeability. Accordingly, the GLO2 downstream product, d-lactate, was decreased dramatically after immune activation, while its enantiomer l-lactate was barely changed (Fig. 4d; Supplementary information, Fig. S4b). Despite activated immune cells having a higher glycolysis rate and a greater afflux towards the glyoxalase system, we found that MGO remained at low levels (Supplementary information, Fig. S4c), indicating the high efficiency of GLO1 in processing MGO. Next, we treated macrophages with S-p-Bromobenzylglutathione cyclopentyl diester (BrBzGCp2) and N, S-bisfluorenylmethoxycarbonyl GSH (DiFMOC-G), the pharmacologic inhibitor of GLO1 and GLO2, respectively (Fig. 4a). The results showed that treatment with DiFMOC-G could dramatically induce SLG accumulation (Fig. 4e), recapitulating changes observed during macrophage activation, while BrBzGCp2 significantly decreased SLG levels (Fig. 4e). These data indicate that SLG accumulation in immune cells is largely due to GLO2 repression after NF-κB activation.

### SLG directly induces d-lactylation modification in immune cells in an enzyme-independent manner

The chemical structure of SLG contains a labile thioester bond that can be attacked by intracellular nucleophiles, such as amidogen on the side chain of lysine residues. This could lead to a nonenzymatic transfer of lactyl moiety from SLG to protein lysine residues, forming a d-lactyllysine modification (K_D-la_) (Fig. 4f). K_D-la_ modification has two types of isomers, l-lactyllysine modification (K_L-la_) derived from l-lactate through enzyme-catalyzed reactions and *N*-ε-(carboxyethyl)-lysine modification (K_CE_) directly induced by MGO (Fig. 4g).^31^ As GLO2 decreased and SLG accumulated during innate immune activation, while no significant increase of l-lactate and MGO was observed, we hypothesized that NF-κB activation-induced SLG accumulation would promote protein d-lactylation, which could be the main type of increased lacK isomers in immune activated cells, contributing to cellular adaptation or feedback regulation of immune activation.

To test this hypothesis, we first examined the level of three isomers of lacK modification in immune cells. The immunoblot assay using K_D-la_ and pan-lacK antibodies showed that K_D-la_ levels were significantly elevated in both activated macrophages and dendritic cells (Fig. 4h; Supplementary information, Fig. S4d). However, there was no obvious change in the levels of K_L-la_, K-Carboxyethyl (K_CE_), and other lysine acylation (Supplementary information, Fig. S4e–g). Consistently, protein modification analysis of proteomic data revealed that lacK modification was most significantly upregulated after macrophage activation (Supplementary information, Fig. S4h) and lacK-specific MS profiling confirmed that lacK peptides were dramatically increased in activated macrophages (Fig. 4i; Supplementary information, Fig. S4i and Table S1). These data indicate that protein lactylation in immune cells was directly induced by SLG accumulation and d-lactylation was the main type of lactylation in activated immune cells.

To verify the effect of SLG on protein lactylation, we directly co-incubated bovine serum albumin (BSA) with SLG in vitro and found that lysine lactylation was induced in an SLG-dose dependent manner (Fig. 4j; Supplementary information, Fig. S5a). We validated this effect by incubating macrophage cell lysates with SLG and obtained the same result (Fig. 4k; Supplementary information, Fig. S5b). Furthermore, the SLG-induced lactylation reaction was further enhanced by heat-denaturing protein pretreatment (Fig. 4j, k; Supplementary information, Fig. S5a, b), which may expose more lysine residues on proteins and inactivate enzymes like GLO2 and delactylases. These data further verified that SLG could directly induce protein d-lactylation modification in an enzyme-independent manner.

Then we used BrBzGCp2 and DiFMOC-G to treat macrophages and found that inhibition of GLO2 increased protein lactylation levels (Fig. 4l; Supplementary information, Fig. S5c), phenocopying changes observed upon macrophage activation, while inhibition of GLO1 impaired activation-induced protein lactylation (Fig. 4m; Supplementary information, Fig. S5d). Consistently, tamoxifen-induced deletion of *Hagh* in macrophages resulted in higher lactylation levels compared with its control counterpart (Fig. 4n; Supplementary information, Fig. S5e), while macrophages from GLO2-overexpression mice showed no increase in lactylation upon treatment of classical activating stimuli (Fig. 4o, p). Furthermore, in GLO2 ARE-sites mutant RAW 264.7 macrophages, as the feedback mechanism of GLO2 downregulation is disrupted, protein lactylation levels did not increase after innate immune activation (Supplementary information, Fig. S5f, g). In addition, the levels of lactylation “erasers” including HDAC1, HDAC2, HDAC3, and SIRT2,^32,33^ or potential lactylation “writers” such as EP300, AARS1, and AARS2, showed no significant change in activated macrophages (Supplementary information, Fig. S5h, i).^19,20^ Also, inhibition of p300 and LDHA did not inhibit the increase of d-lactylation after innate immune activation (Supplementary information, Fig. S5j, k). These data indicate that the enhanced lactylation in activated immunocytes is largely due to GLO2-repression-induced SLG accumulation, rather than changes at the expression level of lactylation “erasers” or “writers”.

### SLG-induced d-lactylation occurs through SN-transfer of the lactyl moiety mediated by an adjacent cysteine residue

**Fig. 5:**
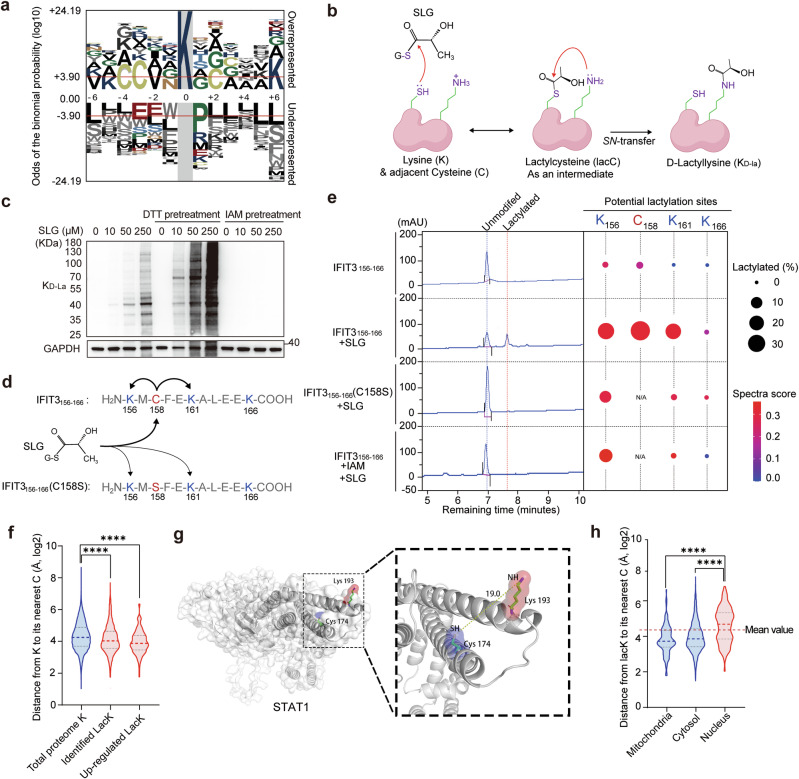
SLG-induced d-lactylation is catalyzed by its adjacent cysteine. **a** Motif analysis of peptide sequences around lacK sites identified in macrophages. **b** Nucleophilic substitutions between SLG and lysine were catalyzed by an adjacent cysteine residue. **c** Immunoblot of K_D-La_ level in BMDM lysates pretreated with DTT (5 mM) or IAM (10 mM), then co-incubated with the indicated concentration of SLG. **d** Sequence of synthesized WT or mutated IFIT3_156-166_ peptide and their presumed reaction mechanism with SLG. **e** LC–MS detection of peptide lactylation (left) and LC–MS/MS detection of lactylation sites (right) on WT or mutated IFIT3_156-166_ peptides pretreated with IAM (10 mM) or not followed by SLG (1 mM) co-incubation. **f** Distance between different lysine (K) ε-amine groups and the spatially closest cysteine (C) β-thiol in these 3D protein structures (KC distance) calculated using Python. **g** 3D structure of STAT1 (PDB 1YVL) and the distance from K193 to its nearest cysteine C174. **h** KC distance of lacK sites we identified with different subcellular localizations.

We performed antibody-enriched lactylome profiling and identified 2255 lacK sites on 901 proteins mostly in activated macrophages (Supplementary information, Table S1). Majority of these proteins prefer to have only one or two lactylation sites (Supplementary information, Fig. S6a), indicating that SLG-induced lactylation is a relatively picky process, in case it becomes a nonselective or even harmful reaction. To explore the mechanism of d-lactylation formation, we analyzed the peptide sequences of these lacK sites. No obvious sequence motif could be identified in these lactylated peptides (Supplementary information, Table S2). However, we found that amino acids with small side chains, such as alanine (A) and glycine (G), were enriched close to the lacK site, while amino acids with large side chains, such as leucine (L), glutamate (E) and proline (P), were generally underrepresented in these peptide sequences (Fig. 5a; Supplementary information, Fig. S6b). The data suggest that for lactylation to occur, lysine residues should be relatively exposed. Interestingly, we found that cysteine (C) residue was significantly enriched around lacK sites in the peptide sequence, e.g., KXC, CXXK, CXXXK. The β-thiol in the side chain of cysteine serves as an active nucleophile, possessing a lower pKa (~8.5) compared to the ε-amine of lysine (~10.5). This characteristic renders it an optimal catalyst under physiological conditions for the acylation of neighboring lysine residues through a reversible S-acylation intermediate. The mechanism above has been observed in the process of lysine acetylation occurring in mitochondria, which is induced by either acetyl-CoA or acetyl-GSH.^34^ We speculated that SLG-induced lactylation is facilitated by *SN*-transfer of the lactyl moiety from an intermediate lactylcysteine (lacC) to lacK (Fig. 5b). To test this hypothesis, macrophage cell lysates were pretreated with the reducing agent dithiothreitol (DTT) to release more cysteines and β-thiols, and then proteins were incubated with SLG. We found that DTT pretreatment dramatically enhanced SLG-induced lactylation (Fig. 5c; Supplementary information, Fig. S6c). In contrast, pretreatment with iodoacetamide (IAM) to alkylate the thiol of cysteine irreversibly, almost blocked SLG-induced lactylation (Fig. 5c), indicating the vital role of cysteine in this reaction.

To obtain direct evidence regarding this reaction, we specifically targeted a lacK site (K161) on the IFIT3 protein, which is close to a cysteine residue (C158). We synthesized this peptide (IFIT3_156-166_) through solid-phase peptide synthesis (SPSS), as well as a cysteine-mutant version (IFIT3_156-166_C158S) (Fig. 5d). These peptides were then incubated with SLG for 4 h and their lactylation levels were measured by high-performance liquid chromatography (HPLC)/MS/MS. As anticipated, the liquid chromatography–mass spectrometry (LC–MS) data showed that incubating wild-type (WT) peptides with SLG resulted in significant lactylation. However, the observed effect was not present in peptides with cysteine-mutant (C158S) or upon IAM-induced cysteine alkylation (Fig. 5e left). MS/MS data revealed that lactylation was only detected on cysteine (C158) and its nearby lysines (K156, K161), while lysine (K166) at the carboxy tail showed no detectable lactylation (Fig. 5e, right; Supplementary information, Fig. S6d). The results support our lactylated peptide sequence data showing that cysteine (C) always lies close to the lacK site (Fig. 5a; Supplementary information, Table S2) and that the distance to cysteine determines lysine lactylation possibility. These data confirm that SLG-induced lactylation in immune cells is largely dependent on its nearby cysteine residue.

However, a cysteine might be distant from the lacK site on the primary sequence but be spatially adjacent in the folded protein structure. Therefore, we used protein structure data from the AlphaFold database^35^ and calculated the distance of each lysine (K) to its spatially nearest cysteine (C) (KC distance for short) on every protein. We discovered that the lacK sites we identified in macrophages have, on average, a shorter KC distance compared to all other lysine residues in the proteome. Additionally, lacK sites that are significantly upregulated after macrophage activation, likely induced by SLG, have an even shorter KC distance (Fig. 5f). This observation was illustrated by intracellular signaling proteins (Supplementary information, Fig. S6e), such as STAT1, whose lacK193 is spatially close to a thiol of C174 (~19 Å) (Fig. 5g). These data suggest that the KC distance of a specific lysine residue and its local accessibility determines its susceptibility to SLG-induced lactylation. It is interesting to note that out of the 2255 lacK sites we identified, the ones located in the cytosol and mitochondria have a much shorter KC distance than those located in the nucleus (Fig. 5h). This suggests that cytosolic and mitochondrial lacK occur in a similar manner in immune cells, which is quite different from lacK in the nucleus.

Collectively, these data suggest that the modification of lacK in immune cells induced by SLG is catalyzed by the adjacent β-thiol of cysteine residues through *SN*-transfer of the lactyl moiety (Supplementary information, Fig. S6f), which also determines the target specificity of the SLG reaction.

### Lactylation in return effectively attenuates immune activation and inflammatory responses

**Fig. 6:**
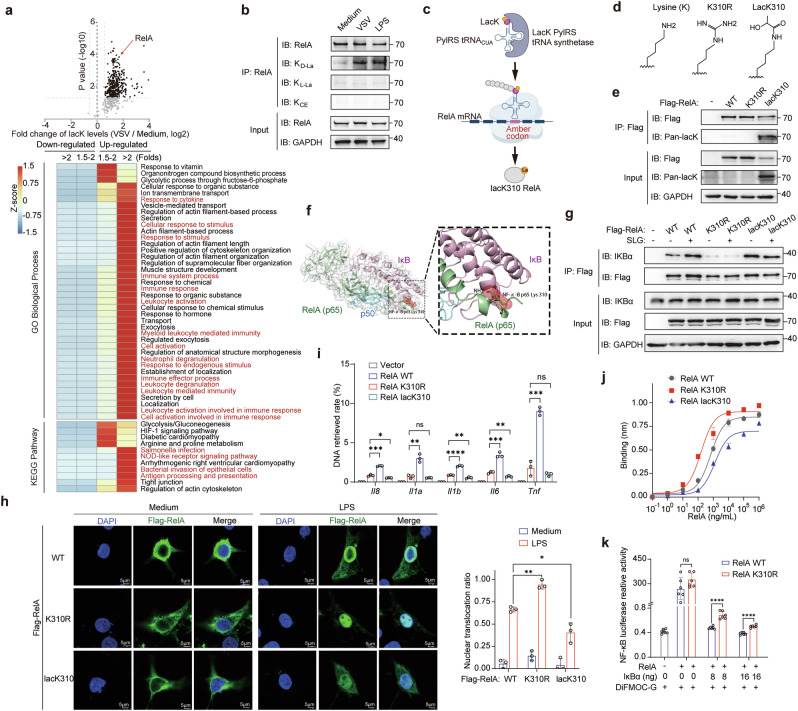
The d-lactylation targets the immune pathway and reduces innate inflammatory signaling. **a** Volcano plot of lacK modification sites identified in macrophages with or without VSV stimulation, *n* = 3 (upper); GO biological process and KEGG pathway enrichment (lower) of genes with upregulated and downregulated lacK levels. **b** Immunoblot of levels of different lactylation isomers of RelA proteins from VSV- or LPS-stimulated macrophages by endogenous immunoprecipitation. **c** Schematic outlines of the use of lacK pyrrolysyl-tRNA synthetase (PylRS) and its cognate tRNA^Pyl^ to mediate site-specific lactylation of RelA protein (lacK310) in mammalian cells. **d** Side chain structure of lysine (K), arginine (R), and lactyllysine (lacK) residues. **e** Immunoblot of WT, site mutant (K310R), and site-specific lactylated (lacK310) RelA proteins purified from human HEK-293T cells. **f** The crystal structure of RelA, IκBα, and p50 complex (PDB 1NFI); enlarged region shows the interface of RelA and IκBα around the K310 residue. **g** Immunoblot detection of indicated proteins co-immunoprecipitated by Flag-tagged WT, K310R mutant, or lacK310 RelA. All RelA proteins were pre-incubated with SLG (1 mM) and then incubated with BMDM cell lysates. **h** Immunofluorescence analysis of WT, K310R, and lacK310 RelA in TLR4-expressing HEK-293T cells co-transfected with IκB stimulated by LPS. Scale bars, 5 μm. **i** ChIP analysis of the binding ability of WT, K310R, and lacK310 RelA with indicated gene promoters. **j** BLI analysis of the binding ability of WT, K310R, and lacK310 RelA with NF-κB-binding DNA fragments. **k** NF-κB luciferase activities in HEK-293T co-transfected with RelA (WT or K310R) and different amounts of IκBα and then treated with DiFMOC-G (0.4 μM). Data were normalized to renilla luciferase, *n* = 6.

Antibody-enriched lactylome profiling revealed that lactylation level in macrophages increased after activation (Fig. 6a, upper panel) and most lactylated proteins (74.6%) were localized in the cytosol (Supplementary information, Fig. S7a), which is consistent with the cytosolic localization of GLO2 and SLG. The Gene Ontology (GO) biological process and Kyoto Encyclopedia of Genes and Genomes (KEGG) enrichment analysis showed that immune activation and inflammatory pathways were significantly enriched, particularly among proteins with greatly increased lactylation levels (fold change > 2.0, VSV/Medium) (Fig. 6a, bottom panel). Several key proteins involved in immune responses were found to have lactylation at highly conserved lysine sites, including RelA, RIG-I, IFIT3, STAT1, and MAPK1 (Supplementary information, Fig. S7b–f). We confirmed that SLG treatment induced lysine lactylation on these proteins in vitro using an immunoblotting assay. We found that RelA was the most significantly lactylated (Supplementary information, Fig. S7g). Using immunoprecipitation experiment, the lactylation on endogenous RelA protein was confirmed to be d-lactylation in VSV- or LPS-stimulated macrophages, while no significant levels of K_L-La_ and K_CE_ were detected on RelA proteins (Fig. 6b).

To unambiguously investigate the functional role of lactylation on RelA (lacK310), we employed a genetic code expansion orthogonal system^36^ to incorporate a lactyllysine into RelA and created a site-specific lactylated protein (lacK310 RelA) in mammal cells (Fig. 6c; Supplementary information, Fig. S7h). Along with this gain-of-function mutant, we also generated a loss-of-lactylation mutant by replacing lysine (K) with an arginine (R) residue (Fig. 6d). We verified the expression and purification of Flag-tagged WT, K310R, and lacK310 RelA using immunoblotting (Fig. 6e). Based on its crystal structure, RelA lactylation on K310 is closely associated with the inhibitory protein IκB (Fig. 6f) and adjacent to a key phosphorylation site linked to transcriptional activation (Serine 311).^37^ Hence, we hypothesized that the modification of lacK310 on RelA could potentially impact the downstream NF-κB signaling pathway. co-immunoprecipitation (Co-IP) assay revealed that the association of RelA with IκB was enhanced by SLG pretreatment or lacK310 modification, and this effect was diminished in the loss-of-lactylation mutant (K310R) (Fig. 6g). Consistently, nuclear translocation of RelA was influenced by lacK310 modification and promoted by K310R mutant (Fig. 6h). We next examined the transcriptional function and found that lacK310 RelA showed decreased binding to target genes in our chromatin immunoprecipitation (ChIP) detection and biolayer interferometry (BLI) kinetic binding assay, while the loss-of-lactylation mutant (K310R) showed stronger binding to target genes than WT RelA (Fig. 6i, j). These data were further confirmed by NF-κB luciferase reporter assay which showed higher transcriptional activity in K310R mutant RelA compared with WT RelA (Fig. 6k). Our results show that SLG-induced d-lactylation of K310 on RelA suppresses NF-κB activation, indicating that cytosolic d-lactylation suppresses immune activation and inflammatory signaling.

### The GLO2 feedback axis coordinates immune activation and inflammation both in vitro and in vivo

**Fig. 7:**
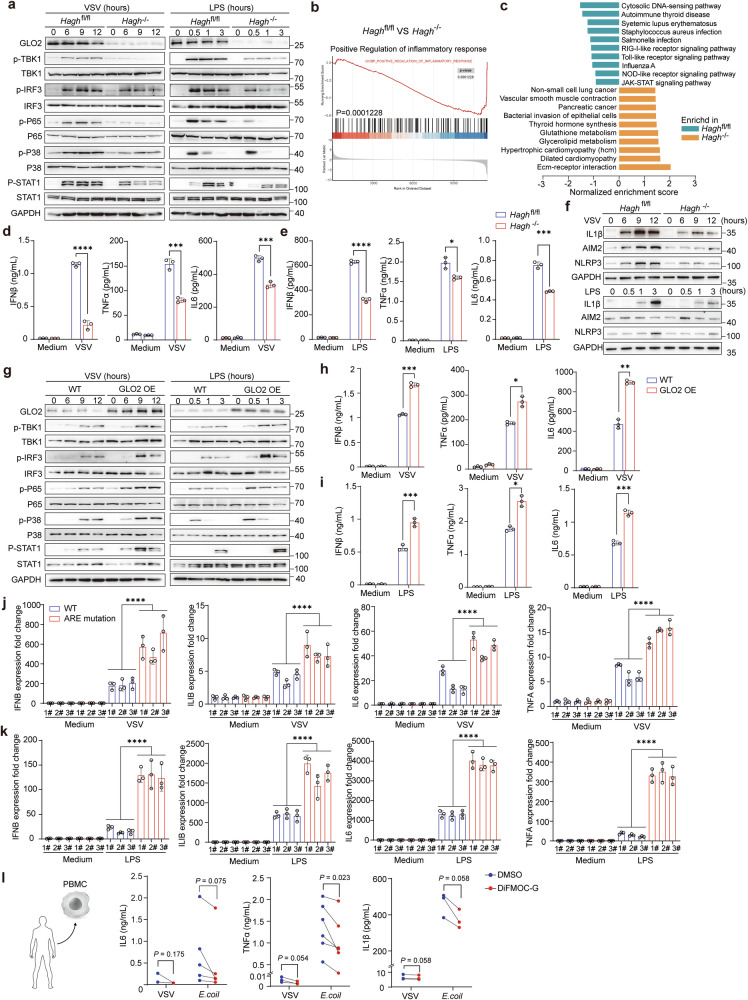
The GLO2 feedback axis regulates inflammatory responses and immune activation in vitro. **a** Immunoblot detection of indicated proteins and their phosphorylation in tamoxifen-treated BMDMs from *Hagh*^fl/fl^ and *Hagh*^fl/fl^*Lyz2*^creERT2^ (*Hagh*^*−/−*^) mice. Cells were stimulated by VSV or LPS for indicated time points. **b**, **c** GSEA analysis of RNA-seq data of *Hagh*^fl/fl^ and *Hagh*^*−/−*^ BMDMs stimulated by VSV for 9 h. **d**, **e** ELISA detection of indicated cytokines in medium supernatants of *Hagh*^fl/fl^ and *Hagh*^*−/−*^ BMDMs stimulated as indicated. **f** Immunoblot detection of indicated proteins in *Hagh*^fl/fl^ and *Hagh*^*−/−*^ BMDMs stimulated by VSV or LPS for indicated time points. **g** Immunoblot detection of indicated proteins and their phosphorylation in BMDMs from WT and GLO2 OE mice. Cells were stimulated by VSV or LPS for indicated time points. **h**, **i** ELISA detection of indicated cytokines in medium supernatants of WT and GLO2 OE BMDMs stimulated as indicated VSV (**h**) and LPS (**i**). **j**, **k** Q-PCR analysis of indicated gene expression in WT or GLO2 ARE-sites mutant RAW 264.7 cells stimulated by VSV (9 h) (**j**) or LPS (2 h) (**k**). **l** Cytometric Bead Array System (CBA) detection of indicated cytokines in medium supernatants of human PBMC stimulated by VSV or LPS with 12 h pretreatment of DiFMOC-G (0.4 μM).

To investigate the role of GLO2 in the immune response and immune disorders, we generated transgenic mice (*Hagh*^fl/fl^*Lyz2*^CreERT2^) where tamoxifen induces the deletion of *Hagh* in myeloid cells (Supplementary information, Fig. S8a). GLO2 was confirmed to be deleted in macrophages with high efficiency (Fig. 1c). We found that GLO2 knockout did not affect the differentiation of macrophages in vitro (Supplementary information, Fig. S8b, c), and had no influence on the intracellular concentration of glutathione and GSH/GSSG redox balance (Supplementary information, Fig. S8d). Our immune blotting results showed that GLO2 genetic deficiency significantly impaired immune inflammatory signaling, especially the TBK1 and NF-κB pathways (Fig. 7a). To comprehensively address the effect of GLO2 on gene expression, we examined the expression profile of *Hagh*^*−/−*^ and *Hagh*^fl/fl^ macrophages using RNA sequencing (RNA-seq). Gene set enrichment analysis (GSEA) revealed that inflammatory response was significantly altered by GLO2 deletion (Fig. 7b). Genes affected by GLO2 were enriched in PRR signaling pathways, autoimmune diseases, and responses to pathogen infection (Fig. 7c). This observation was further validated in macrophages stimulated with different PRR ligands, showing that GLO2 deficiency resulted in lower production of interferons, inflammatory cytokines, and inflammasome effectors at both mRNA and protein levels (Fig. 7d–f; Supplementary information, Fig. S8e, f). In agreement with these results, pharmacologic inhibition of GLO2 and siRNA-mediated GLO2 knockdown showed a similar effect in mouse macrophages (Supplementary information, Fig. S9a–h). However, production of the anti-inflammatory cytokine IL-10 was unaffected by GLO2 inhibition (Supplementary information, Fig. S9i). In contrast, GLO1 inhibition showed the opposite effect, promoting interferon and inflammatory cytokine production in both viral and LPS-stimulated macrophages (Supplementary information, Fig. S9j, k).

To counteract the feedback effect of GLO2 downregulation during immune activation, we created mice overexpressing GLO2 without its 3′UTR (GLO2 OE) (Supplementary information, Fig. S9l). Our observation showed that bone marrow-derived macrophages (BMDM) from these GLO2 OE mice exhibited heightened inflammatory signaling and cytokine production compared to BMDMs from the control group (Fig. 7g–i). More convincingly, GLO2 ARE-sites mutation in RAW 264.7 macrophage, which specifically abolished the TTP-mediated GLO2 feedback axis, led to higher expression of inflammatory cytokines (Fig. 7j, k). The data demonstrate that the GLO2 feedback axis negatively regulates immune activation and inflammation in vitro.

We also examined the effect of GLO2 on human immune cells. GLO2 inhibition in human PBMCs led to decreased expression of interferon and inflammatory cytokines after VSV or *E. coli* stimulation (Fig. 7l; Supplementary information, Fig. S10a, b). As GLO2 also decreased in adaptive immune activation (Fig. 1g), we further investigated the impact of GLO2 on human CD4^+^ and CD8^+^ T-cell activation. Flow cytometry data showed that inhibition of GLO2 reduced IFNγ secretion, indicating that the GLO2 feedback axis plays a role in innate and adaptive immune cells (Supplementary information, Fig. S10c, d).

**Fig. 8:**
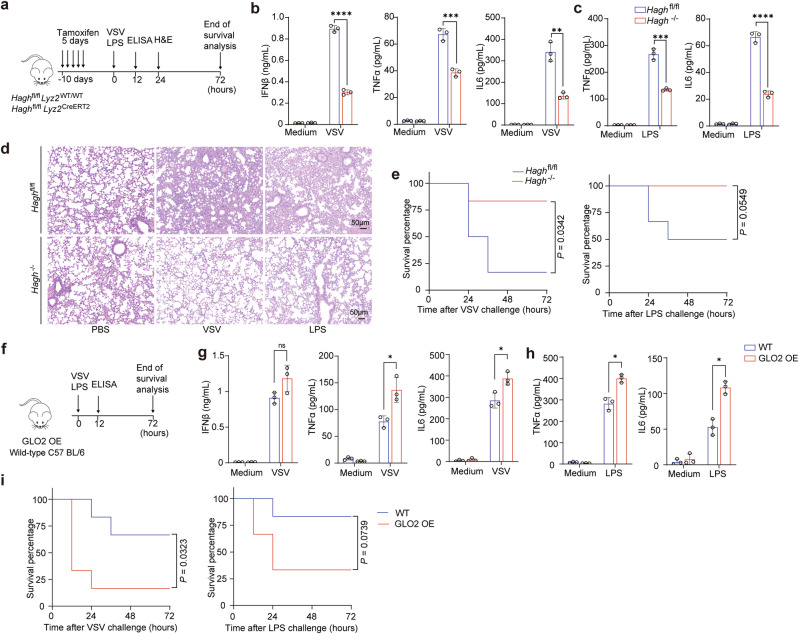
Genetic manipulation of GLO2 alters inflammatory responses and immunopathology in vivo. **a** Experimental schematic outlines of in vivo challenges with VSV and LPS in tamoxifen-induced *Hagh* knockout mice. **b**, **c** ELISA detection of indicated cytokines in the serum of *Hagh*^fl/fl^ and *Hagh*^*−/−*^ mice i.p. injected with VSV (**b**) or LPS (**c**) for 12 h. **d** H&E staining of lung tissues of *Hagh*^fl/fl^ and *Hagh*^*−/−*^ mice after i.p. injected with VSV or LPS for 24 h. Scale bars, 50 mm. **e** Survival of *Hagh*^fl/fl^ and *Hagh*^*−/−*^ mice i.p. injected with VSV or LPS. **f** Experimental schematic outlines of in vivo challenges with VSV and LPS in GLO2 OE mice. **g**, **h** ELISA detection of indicated cytokines in the serum of WT and GLO2 OE mice i.p. injected with VSV (**g**) or LPS (**h**) for 12 h. **i** Survival of WT and GLO2 OE mice i.p. injected with VSV or LPS.

Finally, we verified the effect of the GLO2 feedback axis in vivo. We first verified that the deletion of GLO2 in vivo did not affect the viability and differentiation of myeloid cells and lymphocytes (Supplementary information, Fig. S11a–c). After tamoxifen-induced deletion of *Hagh*, *GLO2*-knockout mice and their littermate controls were challenged with LPS and VSV (Fig. 8a). And the deletion of GLO2 in myeloid cells resulted in decreased levels of interferon and inflammatory cytokines in the spleen, lungs, and serum, consistent with in vitro data (Fig. 8b, c; Supplementary information, Fig. S11d). Notably, mice with GLO2 deficiency in myeloid cells exhibited reduced immunopathological lung injury, as shown by Hematoxylin and Eosin (H&E) staining (Fig. 8d), and had a higher survival rate compared to control mice (Fig. 8e). In contrast, when GLO2 was overexpressed in mice, which blocked the GLO2 feedback axis, there was an increase in the production of interferon and inflammatory cytokines (Fig. 8f–h). This resulted in reduced survival rates during viral and bacterial challenges (Fig. 8i). These findings suggest that the GLO2 feedback axis inhibits inflammation and immune activation in both humans and mice.

### The GLO2 small-molecule inhibitor DiFMOC-G shows a promising therapeutic effect in inflammatory and autoimmune disease models

**Fig. 9:**
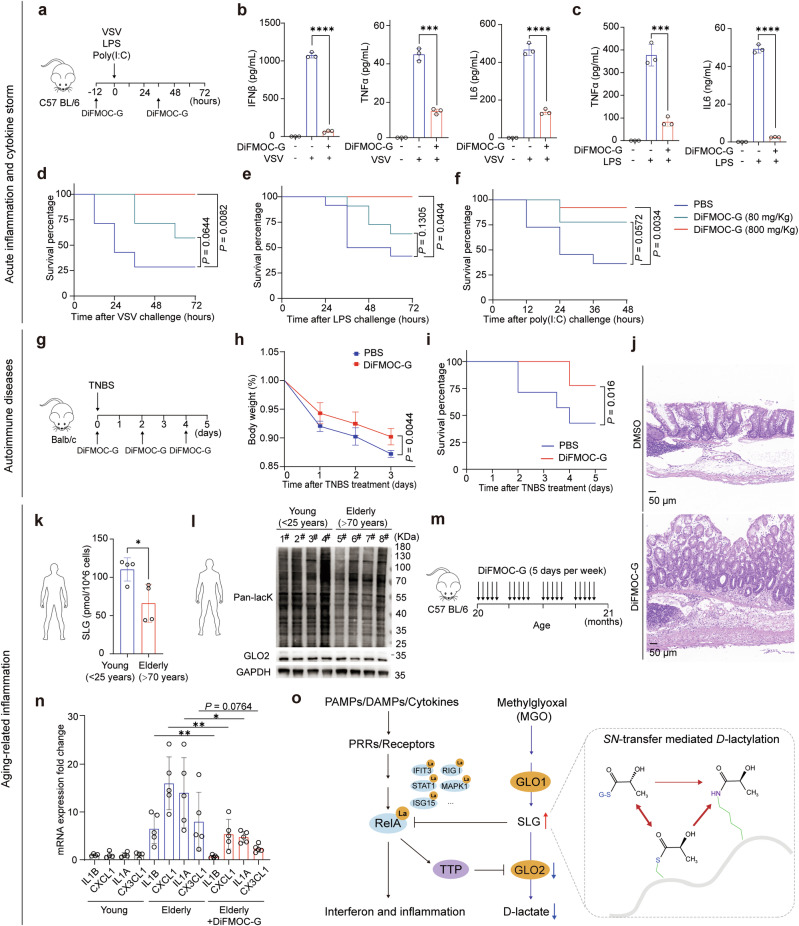
Targeting GLO2 through pharmacologic inhibition demonstrates promising effects in the treatment of inflammatory and autoimmune diseases. **a** Experiment design of acute inflammation and cytokine storm mouse model. **b**, **c** ELISA detection of indicated cytokines in the serum of mice pretreated with DiFMOC-G (800 μg/g) then i.p. injected with VSV (**b**) or LPS (**c**) for 12 h. **d**–**f** Survival of mice pretreated with DiFMOC-G then i.p. injected with VSV (**d**), LPS (**e**), or intravenously (i.v.) injected with Poly (I:C) (**f**). **g** Experimental design for the TNBS-induced colitis model. **h** Body weight of TNBS-induced colitis mice i.p. treated with or without DiFMOC-G. **i** Survival of colitis mice i.p. treated with or without DiFMOC-G. **j** H&E staining of colon tissues from colitis mice i.p. treated with or without DiFMOC-G. Scale bars, 50 mm. **k**, **l** Targeted LC–MS quantification of SLG (**k**) or immunoblot detection of lacK levels (**l**) in VSV-stimulated PBMCs from young (< 25 years old) and elderly (> 70 years old) donors with no obvious metabolic diseases. **m** Experimental design for generating aging-related inflammatory mouse model. **n** Q-PCR analysis of indicated gene expression in the indicated tissues from young (8 weeks) or elderly mice (20 months) i.p. treated with or without DiFMOC-G. **o** Working model of glyoxalase II downregulation feedback orchestrates immune response via its substrate SLG-inducing d-lactylation.

The glyoxalase enzymes have been the focus of pharmaceutical development for inhibitors in metabolic and psychiatric disorders for many years.^38,39^ One such small-molecule enzyme inhibitor, DiFMOC-G, has been identified as the most potent competitive inhibitor of GLO2,^40^ which could be slowly hydrolyzed by GLO2 in vivo and demonstrates excellent pharmacokinetics and safety. Our data so far demonstrate that targeting GLO2 can control inflammatory immune response both in vitro and in vivo. We therefore tested whether DiFMOC-G has any therapeutic effect on inflammatory and autoimmune diseases in vivo. We verified its effect in mouse models with acute inflammation and cytokine storm (Fig. 9a). Intraperitoneal (i.p.) administration of DiFMOC-G attenuated serum levels of inflammatory cytokine and interferon in mice challenged with VSV or LPS (Fig. 9b, c). Importantly, DiFMOC-G treatment, both prophylactic administration and therapeutic administration, promoted mouse survival upon lethal challenges, including infection with VSV or treatment with LPS or poly (I:C) (Fig. 9d–f; Supplementary information, Fig. S12a–d).

Since autoimmune-related genes were significantly repressed by *HAGH* knockout (Fig. 7c) and autoimmune patients had higher GLO2 levels in leukocytes than healthy individuals (Supplementary information, Fig. S12e, f), we then assessed the effect of DiFMOC-G on autoimmune disease models. The mouse model of inflammatory bowel disease (IBD) induced by 2, 4, 6-trinitrobenzene sulfonic acid (TNBS) resembles human autoimmune Crohn’s disease (Fig. 9g). We found that both prophylactic and therapeutic administration of DiFMOC-G attenuated colitis progression and improved the survival ratio of mice (Fig. 9h–j; Supplementary information, Fig. S12g–i).

Low-grade inflammation is a hallmark of senium, a central driver of aging-associated impairment and disease,^41^ and an increasingly common target of pharmacologic interventions.^42^ Interestingly, we found that PBMCs from elderly individuals (> 70 years old) exhibited lower SLG levels (Fig. 9k) and weaker protein lactylation (Fig. 9l) than PBMCs from young adults (< 25 years old). This difference argues that the GLO2/SLG feedback loop declines in elderly individuals and may contribute to low chronic inflammation, the so-called chronic senescence-associated secretory phenotype (SASP).^43,44^ Therefore, we tested the effect of DiFMOC-G in old mice (> 20 months). After administration for one month (Fig. 9m), the level of senescence-associated chronic secretory (SACS) was significantly reduced (Fig. 9n), indicating that GLO2 inhibition using DiFMOC-G may be a promising way to restore immune homeostasis in elderly individuals.

Our data show that GLO2 plays a significant role in immune activation and inflammation in various conditions such as sepsis, infectious diseases, autoimmune disorders, and aging-related inflammation. Additionally, DiFMOC-G, a small-molecule inhibitor of GLO2, shows promising therapeutic effects in models of these diseases.

## Discussion

The role of metabolic alternation and metabolite function has been extensively studied in the field of immunometabolism, with a focus on major energetic pathways such as glycolysis, the Krebs cycle, and lipid metabolism. However, there has been less investigation into the role of their offshoot and other pathways in the metabolic network during immune activation and response. Following a thorough examination of these metabolic enzymes, GLO2 from the glycolysis branching pathway was identified. Its level is decreased by inflammation-induced mRNA decay, and it functions as a positive regulator of immune responses. Mechanistically accumulated SLG upon immune activation directly induces protein lactylation in the cytosol, mainly on immune mediators or effectors, attenuating inflammatory signaling and immune activation as a feedback mechanism (see Fig. 9o).

NF-κB is a widespread transcription factor involved in inflammatory and immune responses, as well as in regulating the expression of many other genes associated with cell survival, proliferation, and differentiation.^45^ In this study, we discovered that NF-κB reduces the level of the metabolic enzyme GLO2 through TTP-induced mRNA decay, which is also a mechanism for resolving inflammatory cytokines. Additionally, the GLO2 substrate SLG induces the d-lactylation of RelA, which in turn dampens NF-κB signaling as a form of feedback. Given the wide-ranging implications of NF-κB in biology and the high conservation of glyoxalase among mammals, the interaction between NF-κB and the glyoxalase pathway may have broader implications in fields such as cancer research, developmental biology, and neurobiology.

Several metabolites from the Krebs cycle, such as itaconate and fumarate, have been shown to directly induce post-translational protein modification in macrophages, T cells, and cancers.^46^ These metabolites all have an active electrophilic chemical structure and tend to attack cysteine residues on their target proteins to perform irreversible Michael additions. This reaction is effectively countered by the presence of the sulfhydryl antioxidant GSH, which is one of the most common molecules in cells. In contrast, SLG from the glyoxalase pathway has a relatively stable chemical structure and does not react with GSH. Its reaction with lysine residues is catalyzed by nearby cysteine residues through reversible nucleophilic substitutions, which provide a moderate reaction mechanism of protein modification in physiological conditions. The target selectivity of SLG in the cytosol is also determined by the cysteine-facilitated mechanism and SLG-induced lactylation is a relatively picky process, to avoid being a universal or harmful reaction. Unlike itaconate and fumarate, which need to translocate from mitochondria to the cytosol or nucleus to modify proteins,^11,12,15^ SLG accumulates in the cytosol and directly modifies cytosolic targets through nucleophilic substitutions, making it a more effective “moonlighting” mechanism than itaconate and fumarate (Fig. 9o). However, as SLG is a harmless and common metabolite in most living organisms, its accumulation-induced lactylation could also be used by certain intracellular bacterial or viral infections to evade immune clearance.

Since the lysine residue contains an active ε-amine group, it is the common target for protein modification that regulates a range of biological processes. Many cytosolic and nuclear lysine modifications have been identified, including ubiquitination, acetylation, methylation, and succinylation.^47,48^ l-lactylation of lysine residues is initially detected on histones in the nucleus of macrophages,^16^ then on MRE11 and p53 in cancer cells,^18,19^ mitochondrial enzymes in myoblast cells, and α-myosin in myocardial cells.^21^ However, SLG-induced lactylation provides another type of lysine modification, d-lactylation, a stereoisomer of l-lactylation. Both types of lactylation interact with other modifications to regulate protein–protein interactions, protein stability, enzyme activities, and cellular localization.^49^ Our data show that d-lactylation of RelA at K310 helps it interact with IκB and attenuates the transcriptional activity of NF-κB (Fig. 6i). This site has been reported to be acetylated by nuclear acetyltransferases P300 and CBP, which could enhance its transcriptional activity.^50^ The lactylation of RelA at K310 probably functions also by hindering its acetylation.

Although we validated that targeting GLO2 could be a potent immune regulator both in vivo and in vitro, unfortunately, we have not identified a cell membrane-permeable SLG surrogate that can probe the physiological function of SLG in immune cells. Such a tool would allow us to further test the proposed mechanism, similar to the use of the itaconate derivative, 4-octyl itaconate (4-OI), to analyze the itaconate function.^51^ Nevertheless, GLO2 inhibition by DiFMOC-G allowed us to increase intracellular SLG accumulation within a physiological range (Fig. 4c, e), making it a more suitable way to investigate the role of GLO2 and SLG in physiological contexts. As a member of the metallo-β-lactamase family, GLO2 has a broad substrate spectrum for glutathione thiol esters with a specific preference for S2-hydroxyacylglutathione derivatives, and SLG is its favorite substrate.^52^ However, we cannot exclude the possibility that GLO2 inhibition by DiFMOC-G might lead to the accumulation of other substrates or unknown side effects. Nevertheless, to our knowledge, SLG is the most abundant and major substrate of GLO2 in vivo and other glutathione thiol esters are present at very low or undetectable levels in immune cells.

Our research shows that the decrease in GLO2 and the buildup of SLG in the cytosol create a feedback loop that regulates NF-κB signaling and inflammatory responses. This process involves SLG-triggered nonenzymatic d-lactylation, catalyzed by a nearby cysteine, which affects inflammatory signaling and immune activation (see Fig. 9o). Our findings not only shed light on a new mechanism of cytosol protein lactylation by the GLO2 substrate SLG but also reveal a previously unrecognized metabolic feedback loop that helps maintain immune homeostasis, through connecting a fundamental and targetable metabolic pathway to the regulation of immune responses.

## Materials and methods

### Cell culture and mice

Mouse peritoneal macrophages were isolated from the peritoneal cavities of mice injected with thioglycolate medium for 3 days and cultured in RPMI-1640 culture medium supplemented with 10% fetal bovine serum (FBS, Gibco). Mouse BMDMs were cultured in DMEM medium with 10% fetal bovine serum (Gibco) and were induced from bone marrow cells by adding 20 ng/mL of recombinant mouse M-CSF (R&D Systems) for 5 days. Mouse bone marrow-derived dendritic cells (DCs) were cultured in RPMI-1640 medium with 10% fetal bovine serum (Gibco) and were induced from bone marrow cells by adding 20 ng/mL recombinant mouse GM-CSF (R&D Systems) and 20 ng/mL recombinant mouse IL-4 (R&D Systems) for 7 days. HEK-293T, THP-1, and RAW 264.7 cell lines were from the American Type Culture Collection (ATCC) and cultured in DMEM medium supplemented with 10% FBS (Thermo Fisher Scientific). All cell lines were tested negative for mycoplasma.

The GLO2 ARE mutation RAW 264.7 cell line was generated using the CRISPR-Cas9 system with the method of homology-dependent repair (HDR). Briefly, the sequence of sgRNA was designed by the online tool CRISPick (https://portals.broadinstitute.org/gppx/crispick/public/), and the design of single-stranded oligodeoxyribonucleotides (ssODNs) complied with the guidelines for donor design from the Geraldine Seydoux lab.^53^ Using Lipofectamine™ 3000 reagent (Invitrogen), RAW 264.7 cell line was co-transfected with ssODN: 5′- CCCTTAGCTTGTGTTATCAAATGTTCTAATGCATATATATAAGAGAAAAAAGGTTTAAAGTATATATTCCCATAACCTCTGCTTATCGACTCTTTATTACCTCTGGCTCTCCCGGTCTATAAC-3′, and pSpCas9(BB)-2A-GFP plasmid (addgene ID: #48138) that was inserted with sgRNA sequence: 5′-ATTCCCATAACCTCTGCTTA-3′. Then monoclonal cells were sorted by Fluorescence Activated Cell Sorting (FACS) system (SH800S, SONY). The sequences of mutation on *Hagh* ARE-sites were sequenced by the Sanger method. The sequencing primers are as follows: F: 5′-ACAGCATGCTGGCGAGACAG-3′; R: 5′-CAGGCATCTCCTTCCCAAAG-3′.

Human PBMCs were isolated from peripheral blood through Ficoll-Hypaque (Mediatech Cellgro) density gradient centrifugation. Blood samples from healthy donors came from the blood centers of Changhai Hospital (Shanghai, China), Changzheng Hospital (Shanghai, China), and Xinhua Hospital of Zhejiang Province (Hangzhou, China). The study was performed in accordance with the declaration of Helsinki and approved by the Ethics Committee of Second Military Medical University, Shanghai, China (no. 20210301-7). PBMCs were cultured in RPMI-1640 medium with 10% FBS (Gibco).

For stimulation of macrophages, DCs, and PBMCs, indicated concentration of LPS (L3755, Sigma-Aldrich), Poly (I:C) (#tlrl-picw, InvivoGen), R848(vac-r848, InvivoGen), C12-iE-DAP (tlrl-c12dap, InvivoGen), HT-DNA (D6898, Merck), β-1,3-glucan (tlrl-curd, InvivoGen), heat-killed preparation of *Candida albicans* (HKCA) (tlrl-hkca, InvivoGen), ATP (HY-B2176, MedChemExpress), nigericin (GC39719, GLPbio), ionomycin (HY-13434, MedChemExpress), and MitoPQ (HY-130278, MedChemExpress) was added to the medium for the indicated time points.

For inhibition experiments, NF-κB inhibitor PDTC (20 μM), TBK1 inhibitor MRT67307 (0.2 μM), JNK inhibitor JNK-IN-8 (1 μM), p38 inhibitor VX-702 (5 μM), AKT inhibitor Akti-1/2 (10 μM), and PKC inhibitor AEB071 (5 μM) were added to the cell culture medium for 1 h and then stimulated as indicated.

For stimulation of macrophages with cytokines, recombined mouse IFNα (10149-IF, R&D systems), IL-6 (406-ML, R&D systems), IL-1β (HY-P7073, MedChemExpress), IFNγ (HY-P700184AF, MedChemExpress), and TNFα (410-MT, R&D systems) were added to the medium for indicated time points. For stimulation of T cells, 100 ng/mL PMA (P8139, Sigma-Aldrich) and 1 μg/mL of ionomycin (HY-13434, MedChemExpress) were added to the medium for the indicated time points. For inhibition of GLO2 or GLO1, the indicated concentration of DiGMOC-G (GA23220, GLPbio), or 5 μM of BrBzGCP2 (HY-136684, MedChemExpress) was added to the medium for the indicated time points. For analysis of mRNA decay, 5 μM of Actinomycin D (HY-17559, MedChemExpress) was added to the medium for the indicated time.

WT C57BL/6 and Balb/c mice were purchased from Sipper BK Experimental Animals (China). *Ifnar*^*−/−*^*, Ifnb*^*−/−*^, and *Il1r*^*−/−*^ C57BL/6 mice were purchased from the Jackson Laboratory. *HAGH* ^fl/fl^*Lyz2*^creERT2^, *Ttp*^*−/−*^, *RelA*^fl/fl^*Lyz2*^creERT2^, GLO2 OE, *Rosa26*^icre^, and K18-hACE2 C57BL/6 mice were purchased or constructed from GemPharmatech Co., Ltd. For tamoxifen-induced knockout of the *GLO2* gene, tamoxifen (HY-13757A, MedChemExpress) was i.p. injected into *HAGH*^fl/fl^*Lyz2*^creERT2^ mice following the standard protocol from the Jackson Laboratory (https://www.jax.org/research-and-faculty/resources/cre-repository/tamoxifen). All mice were housed under specific pathogen-free (SPF) conditions. K18-hACE2 mice were kept in Biosafety Level 3 (BSL-3) housing for the experiments using SARS-COV-2 infection, which was kindly provided by Prof. Zhao Ping. Studies involving experimental animals were approved by the Ethics Committee of the Second Military Medical University, Shanghai, China (no. 20220301-7).

### Pathogens

VSV (Indiana Strain) was amplified by infection of a monolayer of HEK-293T cells. HSV-1 was amplified by infection of a monolayer of Vero cells. Sendai virus (SeV) was propagated in SPF chicken eggs. *E. Coli* (O111:B4) was amplified by culturing in an LB medium. *Listeria monocytogenes* was amplified by culturing in the BHI medium. The SARS-CoV-2 Omicron (B.1.1.529) strain was isolated from a laboratory-confirmed COVID-19 patient. All experiments involving live SARS-CoV-2 were performed in the BSL-3 laboratories at the Second Military Medical University.

### Plasmid constructions and overexpression

Coding sequence (CDS) of mouse IRF3-5D mutant (IRF3(5D)) (Gene ID: 54131), MAPK3(Gene ID: 26417), MAPK p38(Gene ID: 26416), JNK1(Gene ID: 26419), AKT1(Gene ID: 11651), IFIT3 (Gene ID: 15959), MAPK1 (Gene ID: 26413), RelA (Gene ID: 19697), STAT1 (Gene ID: 20846) and IκBα (Gene ID: 18035) were each amplified using high fidelity polymerase KOD Neo (Toyobo) from mouse macrophage cDNAs. And CDS of these genes was cloned into pCDNA3.1/Flag(-) B vector (Life technology). Next, RelA mutations were cloned using site-directed mutagenesis. Vectors carrying relevant coding genes were transfected into HEK-293T cells and used in indicated assays.

### Q-PCR

Total RNA of cells or tissues was isolated with TRIzol (Life technology), and 800 ng of RNA was reverse transcribed with Oligo(dT) or random primers into cDNA with High Efficient Reverse Transcription Kit (Toyobo). The indicated gene was amplified using SYBR Green (Toyobo) by the ABI QuantStudioTM 12 K Flex system. Primer sequences used in Q-PCR were the same as in our previous work.^54^ For analysis of GLO2 isoforms, primers of human cytosolic isoform: F: 5′-GCGCGAGCGCGTTGATTGG-3′; R: 5′-GGCTGGACTGCCGAGCTGC-3′. And primers of human mitochondria isoform: F: 5′-GCAGTCCCCCCACCCACGC-3′; R: 5′-CTCCCTGCCGGGGGCCT-3′. The relative level of RNA expression was normalized to HPRT or ACTB based on the calculation of 2^-ΔΔCt^.

### RNA-seq assay

RNA-seq assays were performed by TIANGEN Co., Ltd. according to TIANGEN’s standard protocol. Briefly, after total RNA extraction and DNase I treatment, the TIANSeq mRNA capture kit (TIANGEN Biotech) was used to enrich eukaryotic total RNAs with Poly-A structure. Then the TIANSeq rapid RNA library construction kit (Illumina platform) (TIANGEN Biotech) was used to construct the library. After RNA fragmentation, cDNA synthesis, end repair, single nucleotide A (adenine) addition, adapter ligation, and PCR library enrichment, construction of the transcriptome sequencing library was completed. During the QC steps, Agilent 2100 Bioanalyzer and ABI StepOnePlus Real-Time PCR System were used in the quantification and qualification of the sample library. Finally, the library was sequenced using Illumina HiSeq2000 or Illumina novaseq 6000, and around 150 bp paired-end sequencing reads were obtained. Differential expression analysis per group was performed using FPKM values. Heatmap and GO/ KEGG/ GSEA pathway enrichment analyses were performed using R-studio, GSEA, and GraphPad Prism 10.0.

### Immunoblotting assay

Cells were lysed with cell lysis buffer (Cat#9803, Cell Signaling Technology) and supplemented with a protease inhibitor cocktail (Calbiochem, La Jolla, CA) for immunoblotting analysis. Protein concentrations of the extracts were measured using the bicinchoninic acid assay (Cat#23225, Thermo Scientific). Different protein samples were loaded onto a gel and subjected to SDS-PAGE, transferred onto nitrocellulose membranes, and then blotted. Anti-GLO2 (AF5944) antibody was from Novus Biologicals, anti-GLO1 (MA1-13029) antibody was from Invitrogen; anti-D- lactyllysine (PTM-1429), anti-l-lactyllysine (PTM-1401RM), anti-lactyllysine (PTM-1401), anti-Carboxyethyllysine (PTM-1701RM), anti-2-hydroxyisobutyryllysine (PTM-801), anti-crotonyllysine (PTM-501), and anti-malonyllysine (PTM-901) antibodies were from PTMbio; anti-phospho-STAT1(Y701) (9167), anti-STAT1 (9172), anti-phospho-TBK1(S172) (5483), anti-TBK1 (3504), anti-phospho-p65(S536) (3033), anti-p65 (8242), anti-IκBα (4814), anti-phosphor-IRF3(S396) (4947), anti-IRF3 (4302), anti-phospho-P38(T180/Y182) (9211), anti-P38 (9212), anti-AlM2 (63660), anti-NLRP3 (15101), anti-IL-1β (12242), and anti-Flag(HRP Conjugate) (86861) antibodies were from Cell Signaling Technology; anti-TTP (12737-1-AP), anti-COXIV (11242-1-AP), anti-Actin (81115-1-RR), and anti-GAPDH (60004-1-Ig) antibodies were from Proteintech.

### scRNA-seq analysis

Four samples from the GSE226488 (GSM7077865 and GSM7077866, labeled as “rest” and “stim”) and GSE243629 (GSM7792046 and GSM7792040, labeled as “HC” and “IA”) datasets in GEO database were analyzed. Expression matrices for all samples were processed in R (version 4.3.1) using the Read10X function from Seurat (version 4.3.0.1). Briefly, for quality control, doublets were removed using “doublets_false” files (for “rest” and “stim”) published on GitHub and the DoubletFinder(version 2.0.3) package (for “HC” and “IA”). Cells with fewer than 3 detected genes, and those with either fewer than 200 or more than 7000 (for “rest”), 6200 (for “stim”), and 4000 (for “HC” and “IA”) detected features were excluded. Additionally, cells exceeding a mitochondrial gene threshold of 14% (for “rest” and “stim”), 15% (for “HC”), and 13% (for “IA”) were filtered out. Each sample was normalized using the SCTransform function. Then samples were integrated via canonical correlation analysis (CCA). Cell clusters were identified based on the UMAP reduced-dimension representation using the FindNeighbors and FindClusters functions, with resolution parameters set at 0.9 for “LPS”, and 0.7 for “IAV”. Finally, the expression of GLO2 was visualized using FeaturePlot and DotPlot.

### Enzyme activity assay of GLO2 and GLO1

The enzyme activity of GLO2 was measured by the Glyoxalase II Activity Assay Kit (Grace Biotechnology, G0145W). Each cell sample (2 × 10^6^) was lysed using extracting solution from the kit, then samples were centrifuged for 10 min at 4 °C,13,000 rpm. Cleared supernatants were transferred to new microcentrifuge tubes and kept at room temperature. According to the manufacturer’s instructions, the absorbance of samples and blanks were read at the reaction time of 2 min and 5 min at a wavelength of 412 nm, and GLO2 activity was calculated according to standard curves.

The enzyme activity of GLO1 was measured using the Glyoxalase I Activity Assay Kit (Sigma-Aldrich, MAK114). Briefly, each cell sample (2 × 10^6^) was lysed by Assay Buffers, and then samples were centrifuged for 10 min at 4 °C,13,000 rpm. Cleared supernatants were transferred to new microcentrifuge tubes. Next, 40 μL of each sample was transferred into two separate microcentrifuge tubes, with one tube as the sample reaction and one tube as the sample blank. The Master Reaction Mix was prepared and the assay was operated according to the instructions of standard protocol. The absorbance of samples and blanks was read at 240 nm (A240), and the GLO1 activity of each sample was calculated according to the standard curves.

### RNA fluorescence in situ hybridization (FISH) and immunofluorescence (IF)

Probes targeting human *GLO2* mRNA were designed using Stellaris® Probe Designer version 4.2 (https://www.biosearchtech.com/support/tools/design-software/stellaris-probe-designer). Probes modified with Cy3 at the 3′ terminus were synthesized by Sangon. Co. Ltd. Then, RNA FISH simultaneous IF was performed according to the protocol for simultaneous IF + Stellaris RNA FISH in adherent cells (LGC, Biosearch Technologies). Briefly, human 293 T cells cultured on poly-l-lysine coated cover slips were fixed with 4% formaldehyde for 10 min, followed by permeabilization with buffers included in the kit. Then cells were resuspended in 1 mL of 70% ethanol for 1 h at 4 °C. After washing, 100 μL of the Hybridization Buffer (SMF-HB1-10, LGC, Biosearch Technologies) containing probe plus (125 nM) and 5 μL of anti-TTP antibody (12737-1-AP, Proteintech) was added onto the parafilm, then incubated in the dark at 37 °C for 12 h. After washing, 1:500 of Alexa Fluor 647 anti-rabbit secondary antibody (Invitrogen, A-21245) was added and incubated in the dark at 37 °C for 30 min followed by staining of nuclei with DAPI (Invitrogen). After washing, the cover slips were mounted with ProLong™ Glass Antifade Mountant (Invitrogen). Images were obtained using a Leica TCS SP8 confocal microscope equipped with a ×63/1.40NA objective. Images were analyzed using the LAS X software version 2.1.2.15022 and Coloc2 from ImageJ (Fiji) 2.14.0 software.

### ChIRP by MS and immunoblotting assay

Probes targeting human and mouse *GLO2* mRNA were designed using ChIRP® Probe Designer version 4.2 (https://www.biosearchtech.com/support/tools/design-software/chirp-probe-designer). Probes modified with biotin-TEG at the 3′ terminus were synthesized by Sangon. co. Ltd. Human THP-1-induced macrophage or mouse PM Cells were stimulated by VSV for 9 h (MOI = 1). 4 × 10^8^ cells were fixed with 4% formaldehyde for 30 min, then cells were lysed and sonicated with 6 mL lysis buffer (50 mM Tris-HCl, pH 7.0, 10 mM EDTA, 2% SDS, 1 mM PMSF, RRI (Takara) 1:50) followed by centrifugation to get clear cell lysates. Cell lysates were mixed with 4 mL of lysis buffer and 10 mL of hybridization buffer (50 mM Tris-HCl, pH 7.0, 750 mM NaCl, 1% SDS, 1 mM EDTA, 15% formamide, 1 mM PMSF, RRI 1:40). After pretreatment with RNase or not, cell lysates were incubated with probes (33 nM) respectively at 37 °C for 12 h followed by adding Dynabeads MyOne Streptavidin C1 (Thermo Fisher Scientific) and incubating for 1 h at 37 °C. After five washes with buffer (2*×* SSC, 0.5% SDS, PMSF 1:100), beads were divided into two parts. One part (1/10 beads) was digested with proteinase K (100 μg/mL, Thermo Fisher Scientific) and subjected to RNA purification using a TRIzol reagent. The other part (9/10 beads) was treated with RNase A (100 μg/mL) at 37 °C for 30 min and then boiled with protein loading buffer for LC–MS/MS and immunoblotting analysis.

For LC–MS/MS analysis, the mass spectrometer detection and data processing were performed by PTMbio according to PTMbio’s standard protocol. Briefly, The proteins in the gel were digested into peptides by adding 10 ng/μL trypsin at 37 °C overnight. The peptides were separated using the EASY-nLC 1200 UPLC system (Thermo Fisher Scientific). After separation by the UPLC system, the separated peptides were analyzed in Orbitrap Exploris 480 with a nano-electrospray ion source. The resulting MS/MS data were processed using the PD search engine (v.2.4).

### RIP (Native-RIP and FA-CLIP)

Native-RIP and FA-CLIP were performed as described previously.^54,55^ Briefly, for native-RIP, cell lysate was harvested with cell lysis buffer (Cell Signaling Technology) directly from 5 × 10^6^ BMDM cells. The TTP–RNA complex was then precipitated with TTP antibody and anti-rabbit magnetic beads (Merck). RNA from TTP–RNA complexes was extracted with the TRIzol reagent. For FA-CLIP, 1 × 10^7^ BMDM cells were fixed with 1% formaldehyde and then lysed with cell lysis buffer (Cell Signaling Technology) and 2% SDS, followed by sonication with a high level until the cell lysate was clarified. The TTP–RNA complexes were then precipitated with TTP antibody and anti-rabbit magnetic beads (Merck). Bead-bound TTP–RNA complexes were eluted with elution buffer (1% SDS, 0.1 M NaHCO3) and de-crosslinked by adding NaCl to 200 mM. Finally, the eluate was digested with Protease K (100 μg/mL) for 30 min and RNA was isolated with TRIzol reagent from beads. Retrieved RNAs were subjected to Q-PCR detection.

### Quantitation of intracellular d-lactate and l-lactate

Intracellular d-lactate and l-lactate were measured by PicoProbe^TM^ d-lactate Assay Kit (Fluorometric) (Abcam, ab174096) and PicoProbe^TM^ l-lactate Assay Kit (Fluorometric, High Sensitivity) (Abcam, ab169557), respectively. Each cell sample (2 × 10^6^) was washed in ice-cold PBS 3 times and then lysed by Assay Buffers. Homogenates were then centrifuged at 12,000 rpm for 10 min at 4 °C and supernatants were collected for subsequent assay. According to the manufacturer’s protocol, the Reaction Mix and Background Control Mix were prepared for each reaction. Next, 50 µL of each sample was added to wells, and 50 µL of the Reaction Mix or Background Control Mix was added and mixed with the contents of each well. The reaction was then incubated for 30 min at 37 °C (d-lactate assay) or room temperature (l-lactate assay) and protected from light. Sample fluorescence (Ex/Em = 535/587 nm) was measured in a microplate reader with kinetics mode, and the concentration of sample d-lactate was calculated according to the standard curves.

### Quantitation of cellular MGO

Cellular MGO was measured by Methylglyoxal Assay Kit (BiotechPack Analytical, BKWB69). Cell samples (2 × 10^6^) were lysed by adding extraction solution from the kit with ultrasonication. Samples were then centrifuged for 10 min at 4 °C, 13,000 rpm. Cleared supernatants were transferred to new microcentrifuge tubes. According to the manufacturer’s instructions, the absorbance of samples and blanks were read at 336 nm, and the concentration of sample MGO was calculated according to standard curves.

### Quantitation of cellular reduced glutathione (GSH) and oxidized glutathione (GSSG)

Cellular GSH and GSSG were measured by Glutathione Assay Kit (S0053, Beyotime) according to the manufacturer’s instructions. Briefly, *Hagh*^fl/fl^ or *Hagh*^*−/−*^ mouse BMDMs were obtained and then lysed by twice quick freezing in liquid nitrogen and heating in a 37 °C water bath. And protein was precipitated and removed by adding buffer M and then centrifuged at 10,000 rpm for 10 min. For the quantitation of total GSH, cell lysates were directly mixed with the reaction buffers (made up according to the manufacturer’s instructions) and then incubated for 5 min at 25 °C; For the quantitation of total GSSG, GSH clear buffer was first added into cell lysates followed by incubated for 60 min at 25 °C, and then lysates mixed with the reaction buffers and incubated for 5 min at 25 °C. The absorbance of samples and blanks was read at 412 nm, and the concentration of sample GSH and GSSG were calculated according to the standard curves.

### Quantitation of SLG by LC–MS

Mouse macrophages or PBMCs were lysed in lysis buffer (0.5% NP-40, 50 mM Tris-HCl, pH 7.4, 150 mM NaCl), then samples were centrifuged for 10 min at 4 °C,13,000 rpm. Protein was precipitated by adding 20% (w/v) 5-sulfosalicylic acid (2% final) and removed via centrifugation at 10,000 rcf for 5 min at room temperature. Next, 100 uL of methanol was added to 100 µL cleared supernatant, then samples were ultrasonicated for 30 min at 4 °C and stood for 30 min at 4 °C. Then samples were centrifuged for 15 min at 4 °C, 12,000 rpm, and prepared for LC/MS detection. For LC separation, 6 µL of clarified supernatant was chromatographed using Waters Acquity UPLC equipped with an Acquity UPLC HSS Amide (1.7 µm, 2.1 mm × 100 mm) at a flow rate of 0.30 mL/min at the temperature of 40 °C with Solvent A (10 mM Ammonium in H_2_O) and Solvent B (Acetonitrile). MS was performed using an AB SCIEX 5500 Qtrap-MS under the following conditions: Ion source: ESI; Curtain Gas: 35 arb; Collision GAS: 9 arb; IonSpray voltage: 4500 V; Temperature: 400 °C; Ion Source Gas1: 45 arb; Ion Source Gas2: 45 arb. For quantitation of SLG, MultiQuant software was used for integration and an SLG standard curve was used for calculation.

### Antibody-enriched lysine lactylation identification (lactylome profiling) by TimsTOF LC–MS/MS

Mouse macrophages (1 × 10^7^) treated with VSV (V) or a medium control (C) were separately harvested and sonicated three times on ice using a high-intensity ultrasonic processor (Scientz) in lysis buffer (8 M urea, 0.5% protease inhibitor, 3 μM TSA and 50 mM NAM). Then lysates were centrifuged at 4 °C 13,000 rpm for 15 min. The supernatant was collected and the protein concentration of the extracts was measured using the bicinchoninic acid assay. Next, trypsin digestion, enrichment of lactylation peptides, and mass spectrometer detection were performed by PTMbio according to PTMbio’s standard protocol.

Briefly, for trypsin digestion, 2% trypsin was added for overnight digestion. Peptides were reduced with 5 mM dithiothreitol for 30 min at 56 °C and alkylated with 11 mM iodoacetamide for 15 min at room temperature in darkness. For enrichment of lactylated peptides, tryptic peptides were dissolved in NETN buffer (100 mM NaCl, 1 mM EDTA, 50 mM Tris-HCl, 0.5% NP-40, pH 8.0), and were incubated with anti-Lactyllysine antibody-conjugated agarose beads (PTM-1404, PTMbio) at 4 °C overnight. After elution of peptides, the peptides were desalted with C18 ZipTips (Millipore) according to the manufacturer’s instructions. For mass spectrometer detection, peptides were separated on a nanoElute UHPLC system (Bruker Daltonics), followed by timsTOF Pro (Bruker Daltonics) mass spectrometry. Precursors and fragments were analyzed at the TOF detector with an MS/MS scan range from 100 to 1700 m/z. Precursors with charge states 0–5 were selected for fragmentation.

For identification and relative quantification of lactylation sites, the MS/MS spectra data were analyzed by MaxQuant software (v.1.6.15.0).^56^ Tandem mass spectra were searched against the mouse SwissProt database. Trypsin/P was specified as a cleavage enzyme allowing up to 2 missing cleavages. The mass tolerance for precursor ions was set as 20 ppm in the first search and 5 ppm in the main search, and the mass tolerance for fragment ions was set as 0.02 Da. Carbamidomethyl on Cys was specified as a fixed modification. Acetylation on protein amino-terminus, oxidation on Met, and Lactylation on Lys were specified as variable modifications. FDR was adjusted to < 1%. Label-Free quantification mode was set as LFQ. The “LFQ min. ratio count” was set as 2. The “LFQ min. number of neighbors” was set as 3, and the “LFQ max. Number of neighbors” was set as 6. The “Label min. ratio count” was set as 2. Peptides for quantification were set as “Unique + Razor”. Acetylation on protein amino-terminus and oxidation on Met were specified as modifications used in protein quantification, and unmodified counterparts were discarded for protein quantification.

Based on the signal intensity value of each peptide in different samples from searching results, the relative quantitative value of the lactylation site is calculated through the following steps: First, the signal intensity (I) of the modified peptide in different samples is transformed to obtain the relative quantitative value (R) of the modified peptide in different samples. The calculation formula is as follows: (i represents the sample, j represents the peptide)

$${R}_{{ij}}={I}_{{ij}}/{Mean}({I}_{j})$$

Furthermore, the relative quantitative value of the lactylation site is divided by the relative quantitative value of the protein corresponding to the modification site to eliminate the influence of protein expression levels in different samples.

### Lysine acylation analysis of the proteome data from mouse macrophages

Protein extraction and trypsin digestion protocols are described above in the section “Antibody-enriched lysine lactylation identification by TimsTOF LC–MS/MS”. The peptides were desalted with C18 ZipTips (Millipore) according to the manufacturer’s instructions. Then peptides were separated into 30 fractions by HPLC and analyzed using a Q-Exactive Orbitrap Mass Spectrometer. For analysis of different types of lysine acylation sites, the MS/MS spectra data were processed using pFind 3.1 software.^57^ Tandem mass spectra were searched against the mouse SwissProt database. Trypsin/P was specified as a cleavage enzyme allowing up to 3 missing cleavages. The mass tolerance for precursor and fragment tolerance was set to 20 ppm. Carbamidomethyl on Cys was specified as a fixed modification and indicated types of lysine acylation on Lys were specified as variable modifications. FDR was adjusted to < 1%, open search was set as none, and quantification was set as none.

### In vitro SLG-induced protein d-lactylation reactions

Different concentrations of SLG (L7140, Sigma-Aldrich) (0 mM, 0.05 mM, 0.25 mM, 1 mM, 1.25 mM, 5 mM, 25 mM) were prepared in TBS buffer (50 mM Tris-Cl, pH 8.0, and 150 mM NaCl) solutions. For reactions examining BSA lactylation, 2 μg/μL solutions of fatty acid-free BSA (A1933, Sigma-Aldrich) were made in TBS. Samples were heated at 95 °C for 15 min for denaturation while control samples were placed on ice for 15 min. When all samples had returned to room temperature, 10 uL of SLG was added to 40 uL of each BSA solution to a final concentration of 0 mM, 0.01 mM, 0.05 mM, 0.2 mM, 0.25 mM, 1 mM, or 5 mM respectively, and samples were incubated for 4 h at 37 °C at 400 rpm in an Eppendorf Thermomixer. Next, the samples were boiled with protein loading buffer for 5 min and then subjected to SDS-PAGE. The level of lactylation was detected by immunoblotting. The input BSA was confirmed by Coomassie brilliant blue R-250 staining.

To measure macrophage lysate protein lactylation, 5 × 10^6^ mouse macrophages were harvested and lysed in 600 uL Lysis Buffer (0.5% NP-40, 50 mM Tris-Cl, pH 8.0, and 150 mM NaCl) supplemented with Protease inhibitor. 40 uL of supernatants were transferred to different tubes according to different reaction conditions. For the control group, samples were placed on ice. For heat denaturation groups, samples were heated at 95 °C for 15 min. For the DTT-pretreated group, samples were reduced with 5 mM dithiothreitol (A620058, BBI) for 30 min at 56 °C and then placed on ice. For the cysteine alkylation group, samples were reduced with 5 mM dithiothreitol for 30 min at 56 °C and then alkylated with 10 mM iodoacetamide (A600539, BBI) for 15 min at room temperature in darkness. SLG co-incubation and detection of lactylation were the same as for the BSA experiment above.

### Assay of SLG-induced d-lactylation on peptides in vitro

WT or mutated peptides from mouse IFIT3 were synthesized by Genscript, and 20 ug of each peptide was dissolved in 200 μL TBS (50 mM Tris-Cl, pH 8.0, and 150 mM NaCl) solutions respectively for each reaction. For the SLG treatment group, peptides were treated with 1 mM of SLG (L7140, Sigma-Aldrich) for 4 h at 37 °C at 400 rpm in an Eppendorf Thermomixer. For the group of cysteine alkylation, peptides were alkylated with 10 mM iodoacetamide (A600539, BBI) for 15 min at room temperature in darkness and then treated with 1 mM of SLG for 4 h at 37 °C at 400 rpm in an Eppendorf Thermomixer. Next, peptide lactylation was detected by LC–MS (Agilent 1200 system), and lactylation sites were identified by LC–MS/MS (Q-Exactive HF-X, Thermo Fisher). MS/MS spectra were analyzed using pFind 3.1 software.

### Distance calculation of lysine ε-amine group to the spatial nearest cysteine β-thiol in the whole proteome

The PDB structure files of the entire proteome were downloaded from the Alphafold database. Python was then used to analyze the PDB files of a given protein. Briefly, we used an “For” loop to walk through each row of the PDB files to find the position of a Lys. The spatial position of the ε-amino group was found in information for the specific Lys. Then, we similarly defined the positions of all Cys groups in the protein and their sulfhydryl groups. The spatial distance between the specific ε-amino and the sulfhydryl was calculated, and the shortest distance and its corresponding Cys were recorded. This distance was defined as the KC distance to the specific Lys site. Codes of KC distance analysis in Python can be obtained upon request to the corresponding author.

### Expression of a site-specific lactylated protein by genetic code expansion with an orthogonal system

The pEF1-Mm (LacK PylRS) plasmid expressing lacyl-tRNA synthetase and tRNA pairs was kindly provided by Prof. Tao Peng (School of Chemical Biology and Biotechnology, Peking University Shenzhen Graduate School), and Flag-tag of lacyl-tRNA synthetase protein was changed to Myc-tag. For expression of site-specific lactylated RelA protein, briefly, a plasmid expressing Flag-RelA containing an amber TAG codon mutation in the K310 site and pEF1-Mm (LacK PylRS) plasmid were co-transfected into HEK-293T cell by JetPei DNA transfection reagents (101000020, PolyPlus). The indicated concentration of lactyllysine was then added to the cell medium. After transfection for 24 h, cells were harvested and lysed in 400 uL Cell Lysis Buffer supplemented with protease inhibitor (539134-1SET, Merck). After centrifugation (13,000 rcf, 10 min, 4 °C), expression of lactylated RelA was detected by immunoblotting, and lysate supernatants were incubated with anti-Flag M2 magnetic beads (M8823, Merck) (rotating at 4 °C for 4 h). Lactylated RelA proteins were eluted by adding 100 ng/µL 3× Flag peptides and then used for the indicated experiments.

### ChIP

2 × 10^7^ HEK-293T cells overexpressing WT or mutated RelA were crosslinked with 1% formaldehyde for 15 min at 37 °C. Crosslinking reactions were quenched with 0.125 M glycine for 5 min at room temperature. Cells were lysed (1% SDS, 50 mM Tris-HCl, pH 8.0, 5 mM EDTA, and protease inhibitors) and sonicated to obtain DNA fragments of ~300–500 bp in length on average. Samples were then centrifuged at 14,000 rpm for 10 min at 4 °C. The supernatant was diluted (20 mM Tris-HCl, pH 8.0, 2 mM EDTA, 1% Triton X-100, 150 mM NaCl, and protease inhibitors) and then incubated with 30 μL Flag M2 magnetic beads (M8823, Merck) overnight at 4 °C. Beads were then washed sequentially with TSE I (0.1% SDS, 20 mM Tris-HCl, pH 8.0, 2 mM EDTA, 1% Triton X-100, 150 mM NaCl and protease inhibitors), TSE II (0.1% SDS, 20 mM Tris-HCl, pH 8.0, 2 mM EDTA, 1% Triton X-100, 500 mM NaCl and protease inhibitors), LiCl buffer (10 mM Tris-HCl, pH 8.0, 1 mM EDTA, 0.25 mM LiCl, 0.1% NP-40 and 1% deoxycholate sodium) and TE (10 mM Tris-HCl, pH 8.0 and 1 mM EDTA, pH 8.0). The mass of bound proteins in beads was detected by SDS-PAGE and immunoblotting and then quantified by ImageJ. Beads binding the same mass as the protein–DNA complex were eluted with 400 μL of fresh elution buffer (25 mM Tris-HCl, pH 8.0, 10 mM EDTA, and 0.5% SDS) at 65 °C for 15 min. Crosslinking of protein–DNA complex and whole-cell extract-DNA was reversed by overnight incubation at 65 °C with 200 mM NaCl. The retrieved rate of immunoprecipitated DNA was analyzed by qRT–PCR.

### BLI kinetic binding studies

NF-κB DNA sequence with 5′ biotin-TEG modification was synthesized by Sangon Biotech Co. (Shanghai, China). Flag-tagged WT, K310R, and K310L RelA proteins were each overexpressed in HEK-239T cells and immunoprecipitated using anti-Flag M2 magnetic beads (M8823, Merck). Proteins were eluted with 3*×* Flag peptides (200 μg/mL) and concentrated by ultrafiltration. The protein concentration was quantified using a BCA assay and then adjusted to the same concentration for the next BLI kinetic binding assays. BLI analysis was performed using the Octet Red 96 system (Starorius). Briefly, biotin-modified NF-κB sequences were loaded onto Streptavidin Biosensors at 1000 rpm for 180 s. WT or K310L RelA proteins diluted to the indicated concentrations were associated with loaded sensors at 1000 rpm for 300 s and dissociated for another 300 s. The data were analyzed using Octet Data Analysis Software version 9.0.0.14.

### Co-immunoprecipitation and endogenous co-immunoprecipitation

Control vectors, WT, or mutated Flag-RelA expressing vectors were transfected in HEK-293T cells (2 × 10^6^) by JetPei DNA transfection reagents (101000020, PolyPlus). After transfection for 24 h, cells were harvested and lysed by 400 uL Cell Lysis Buffer supplemented with protease inhibitor (539134-1SET, Merck). After centrifugation (13,000 rcf, 10 min, 4 °C), lysate supernatants were incubated with 20 uL anti-Flag M2 magnetic beads (M8823, Merck) (rotating at 4 °C for 4 h). Beads were then washed three times in buffer and co-incubated with 50 uL 1 mM SLG in the reaction buffer (150 mM NaCl, 50 mM Tris-HCl, pH 8.0) or buffer alone (control) with rotation at 37 °C for 3 h. After SLG incubation and washing, the mass of binding Flag-RelA proteins was detected and quantified by immunoblotting followed by imageJ analysis. According to the result of quantitation, the beads binding with the same mass of three types of RelA proteins were co-incubated respectively with 400 uL of macrophage lysate supernatants (rotating at 4 °C for 4 h). Beads were washed with wash buffer three times, boiled in protein loading buffer for 5 min, and subjected to SDS-PAGE and immunoblotting analysis.

For endogenous immunoprecipitation of macrophage RelA proteins, mouse macrophages (1 × 10^7^) treated with VSV, LPS, or a medium control were separately harvested using 1 mL cell lysis buffer (Cell Signaling Technology). Then lysates were centrifuged at 4 °C 13,000 rpm for 15 min. The supernatant was collected and the protein concentration of the extracts was measured using the bicinchoninic acid assay. To reduce nonspecific binding, lysates were incubated with 500 uL protein A magnetic beads for 2 h. Next, 10 μL of RelA (NF-κB p65) antibodies (8242, Cell Signaling Technology) were added separately into lysates and incubated overnight at 4 °C, and then protein–antibody complex was incubated with 200 uL protein A magnetic beads at room temperature for 30 min. Beads were washed with wash buffer three times, boiled in protein loading buffer for 5 min, and subjected to SDS-PAGE and immunoblotting.

### Dual-luciferase reporter assays

HEK-293T cells (1 × 10^5^) were cultured in 96-well plates. 20 ng of WT or mutated RelA vector, 100 ng of NF-κB pGL4-Photinus pyralis reporter vector, 10 ng of pGL4-Renilla reniform control vector, and 8 ng or 16 ng of IκBα vector was mixed according to the experimental design. The vector mixture was transfected into HEK-293T cells by JetPei DNA transfection reagents (101000020, PolyPlus). After transfection for 12 h, 0.4 μM of DiFMOC-G was added to each well. After transfection for 36 h, cells were harvested and lysed using Passive Lysis Buffer (E1941, Promega) with shaking for 400 rpm at room temperature for 1 h. Fluorescence intensity was detected using the Dual-Luciferase® Reporter Assay System (E1910, Promega) according to the manufacturer’s instructions.

### Enzyme-linked immunosorbent assay (ELISA)

The concentrations of mouse IFNβ, IL-6, or TNFα in cell culture supernatants or serum were measured using the IFNβ cytokine specific VeriKine ELISA kit (PBL Interferon Source), IL-6 Quantikine ELISA kit (R&D Systems), or TNFα Quantikine ELISA kit (R&D Systems) respectively, according to the manufacturer’s instructions.

### Cytometric Bead Array System (CBA) detection of cytokines

The concentrations of human IL-6, TNFα, or IL-1β in the PBMC cell medium were measured using Human IL-6 Flex Set (558276, BD), Human TNF Flex Set (558273, BD), and Human IL-1β Flex Set (558279, BD), respectively, and detected with the ID7000 Spectral Cell Analyzer (SONY) according to the manufacturer’s instructions. The statistical significance of comparisons between the two groups was determined with a ratio-paired *t*-test.

### Flow cytometry assay

To examine the viability and differentiation of mouse BMDM, *HAGH*^fl/fl^ or *HAGH*^*−/−*^ BMDMs were harvested on culture day 7. Macrophage markers were stained with fluorophore-conjugated CD11b (101235, Biolegend) and F4/80(123115, Biolegend). To examine the proportion and quantity of immune cell subsets in mouse spleen, *HAGH*^fl/fl^ or *HAGH*^*−/−*^ solenocytes were harvested and then stained with fluorophore-conjugated CD11b (101235, Biolegend), CD3 (553062, BD), CD4 (563151, BD), and CD8 (557959, BD).

For the assay of human T-cell activation, Human PBMCs (3 × 10^5^) were cultured in 96-well plates, and 0.4 μM of DiFMOC-G was added to each well for 12 h. Then 100 ng/mL PMA (P8139, Sigma-Aldrich), 1 μg/mL of ionomycin (HY-13434, MedChemExpress), and 3 μg/mL of Brefeldin A Solution (00-4506-51, Invitrogen) were added to the medium for 5 h. For the flow cytometry assay, T-cell markers were stained with fluorophore-conjugated CD3 (561806, BD), CD4 (557852, BD), and CD8 (562282, BD) antibodies. After cell fixation and permeabilization (554714, BD), cytoplasmic IFNγ was stained with fluorophore-conjugated antibody (551385, BD). All samples were assayed with the BD LSR Fortessa flow cytometer, and the data were analyzed by FlowJo software (10.8.1).

### siRNA knockdown of GLO2 and TTP

10 nM siRNA was transfected into the indicated cells using the INTERFERin® reagent (Polyplus) according to standard procedures. 48 h after transfection, cells were detected or treated as indicated. The mouse GLO2-specific sequence (designed and synthesized by RIBOBIO) 5′-UUU CGC AGC UUC UAU UUC C-3′ was used for siRNA-1, 5′-AGU UUC UCU UGA AUG GCA G-3′ was used for siRNA-2. 5′ -UUG UAC AUC UCA UCU GCA G-3′ was used for siRNA-3. The mouse TTP-specific sequence (designed and synthesized by GenePharma) 5′-GCU CCU UCA AGU UGU GAA ACG-3′ was used for siRNA-TTP. The untargeted sequence 5′-UUC UCC GAA CGU GUC ACG U-3′ was used as a negative control.

### SARS-CoV-2 infection mouse model

K18-hACE2 mice, aged 6 weeks, were intranasally injected with 10^5^ TCID_50_ of SARS-CoV-2 Omicron (B.1.1.529). After infection for 3 days, the mice were euthanized and tissues (spleen, lung, and brain) were collected. Total RNA was extracted with TRIzol (Life technology), and RNA was reverse transcribed into cDNA with the High Efficient Reverse Transcriptase Kit (Toyobo). Levels of RNA expression were quantified by Q-PCR. All experiments were performed in BSL-3 laboratories and approved by the Institutional Committee for Animal Care and Biosafety of the Second Military Medical University.

### Inflammatory and acute infection mouse model

For the experiments on *GLO2*-knockout mice, 8-week-old C57 B6/L mice were i.p. injected with VSV (1 × 10^7^ pfu/g) or LPS (80 μg/g). 12 h later, whole blood was collected from the orbital venous plexus. H&E staining of lung tissue was performed 24 h after injection. For the survival assay, mice were kept for 72 h after challenging and then euthanized, *n* = 6.

For DiFMOC-G treatment, 80 or 800 mg/kg of DiFMOC-G was i.p. injected into 8-week-old C57 B6/L mice 12 h before the infection. Then mice were i.p. injected with VSV (1 × 10^7^ pfu/g), LPS (80 μg/g), or i.v. injected with Poly (I:C) (20 μg/g). Whole blood was collected 12 h after the last administration from the orbital venous plexus. For the survival assay, mice were kept for 72 h after challenging and then euthanized. The number of mice used in each group is listed as follows. For VSV challenge, *n* = 7; for LPS treatment, *n*_DMSO_ = 12, *n*_80mg/kg DiFMOC-G_ = 11, *n*_800mg/kg DiFMOC-G_ = 5; for of Poly (I:C) challenge, *n*_DMSO_ = 5, *n*_DiFMOC-G_ = 7. Survival analysis was determined using the Log-rank (Mantel-Cox) test.

### TNBS-induced colitis mouse model

6-week-old Balb/c mice were used for the TNBS-induced colitis model. First, each mouse was weighed and marked before other operations. Then the mouse was anesthetized by i.p. injection of Pelltobarbitalum Natricum (50 mg/kg), and a catheter was fitted to a 1 mL syringe and 2.5% TNBS solution (MB5523, Meilunbio) (water: alcohol = 1:1 solution) was filled. The catheter was inserted into the colon 4 cm proximal to the anus, and 100 mL of TNBS solution was slowly administered into the colon lumen. The catheter was removed gently from the colon and the mouse kept its head down in a vertical position for 60 s. Then, 800 mg/kg of DiFMOC-G in normal saline solution or the same volume of normal saline was i.p. injected into mice, and the same dose of DiFMOC-G was administered once every 48 h. Body weights, H&E staining of colon tissues, and survival ratio were analyzed at the indicated time point after TNBS treatment. Mice were euthanized 5 days after TNBS treatment.

### Statistical analysis

All statistical analyses were performed using GraphPad Prism 9.0 software. Error bars in Q-PCR experiments, cytokine levels, metabolite levels, and enzymatic activity results represent the standard deviation of three or more independent experiments. Data from imaging experiments are representative of three or more independent experiments. Unless noted otherwise, immunoblots are representative of at least three independent experiments. For in vivo data and human data, points represent individual mice or samples from individual donors. The statistical significance of comparisons between the two groups was determined with Student’s *t*-test. The significance of different groups was determined using a one-way or two-way ANOVA as appropriate. Survival analysis was determined with the Log-rank (Mantel-Cox) test. *P*-values of less than 0.05 were considered statistically significant. ns (non-significant) means *P* > 0.05; *P < 0.05; **P < 0.01; ***P < 0.001; ****P < 0.0001.

## Supplementary information

Supplementary information, Fig. S1

Supplementary information, Fig. S2

Supplementary information, Fig. S3

Supplementary information, Fig. S4

Supplementary information, Fig. S5

Supplementary information, Fig. S6

Supplementary information, Fig. S7

Supplementary information, Fig. S8

Supplementary information, Fig. S9

Supplementary information, Fig. S10

Supplementary information, Fig. S11

Supplementary information, Fig. S12

Supplementary information, Table S1

Supplementary information, Table S2

## Data Availability

Our transcriptome RNA-seq data from macrophages with or without SeV stimulation are deposited in GEO under the accession number GSE230756 and transcriptome RNA-seq data from WT or *GLO2*-knockout macrophages with or without VSV stimulation are deposited in GEO under the accession number GSE248885. Mass spectrometry proteomics data are deposited in the ProteomeXchange Consortium under the accession numbers PXD042171, PXD046975, and PXD047003.
