# Supplementary material for: Nonenzymatic lysine d-lactylation induced by glyoxalase II substrate SLG dampens inflammatory immune responses
Source: Cell Res. 2025 Jan 6;35(2):97–116. doi: 10.1038/s41422-024-01060-w (PMC11770101; doi:10.1038/s41422-024-01060-w)
Supplement: Supplementary file 1 — Supplementary information, Fig. S1 [file 41422_2024_1060_MOESM1_ESM.pdf]

Supplementary information, Fig. S1

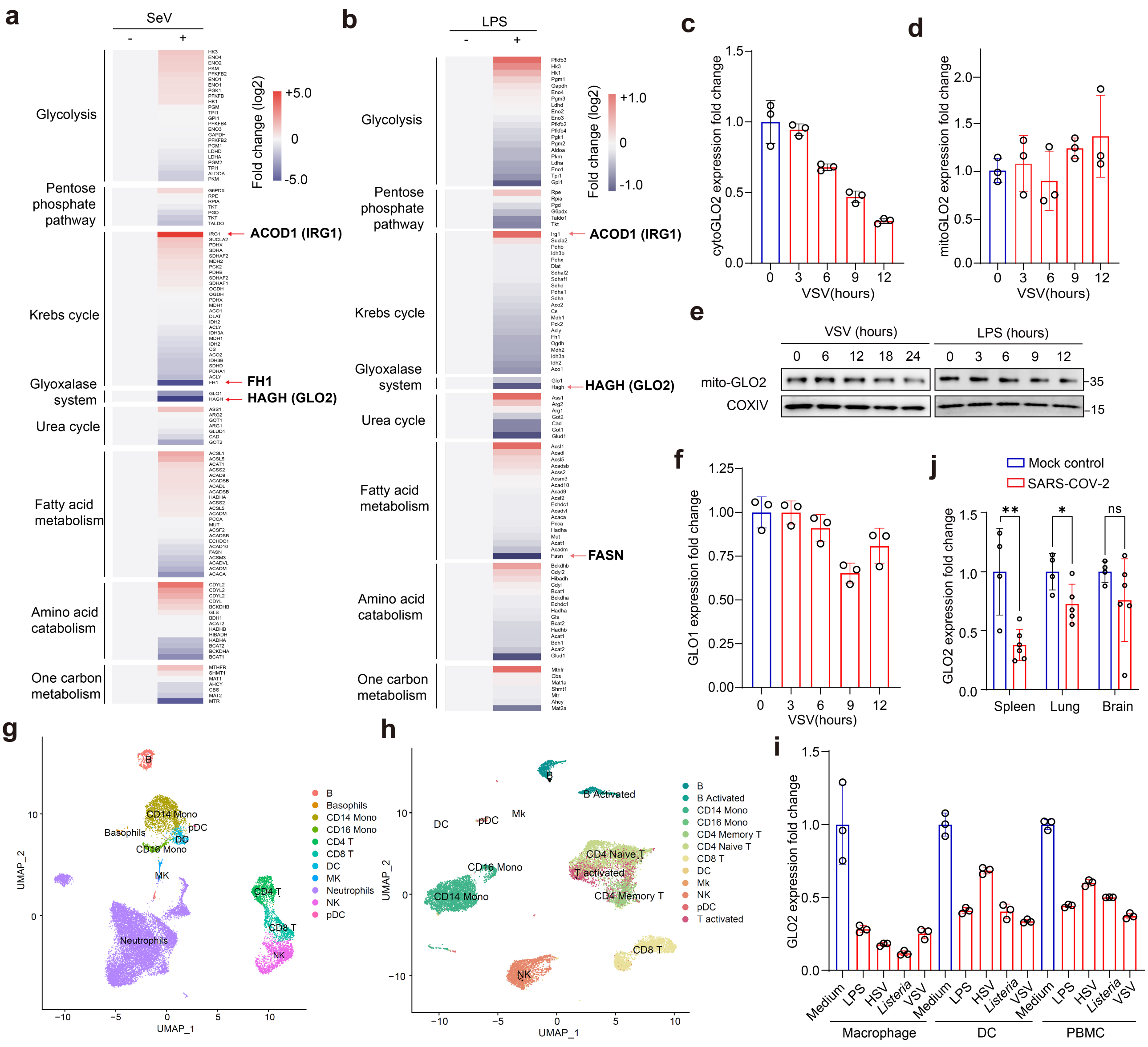

Fig. S1 GLO2 is downregulated in activated immune cells. a, b, Heatmap of RNA-seq data profiling of metabolic enzymes in mouse macrophages stimulated by Sendai virus (SeV) (12h, MOI = 1) **(a)** or LPS (6h, 100 ng/mL) **(b)**. The control group is set to a value of 1**. c, d,** Q-PCR analysis of GLO2 isoforms expressed on cytosolic **(c)** or mitochondria **(d)** in human PBMCs stimulated with VSV for indicated times. **e,** Immunoblot detection of GLO2 proteins isolated from mitochondria in mouse BMDM stimulated with VSV or LPS for indicated times. **f.** Q-PCR analysis of GLO1 mRNA in BMDMs stimulated with VSV for indicated times. **g, h,** UMAP clustering of scRNA-seq of different immune cells in human PBMCs infected with influenza A virus

(GSE243629) **(g)** or stimulated by LPS (GSE226488) **(h)**. **i,** Q-PCR analysis of GLO2 in indicated cells stimulated as indicated. **j**, Q-PCR analysis of GLO2 mRNA in indicated tissues from K18-HACE2 mice intranasally injected with the SARS-CoV-2 Omicron (B.1.1.529) variant for 3 days.
