# Supplementary material for: Nonenzymatic lysine d-lactylation induced by glyoxalase II substrate SLG dampens inflammatory immune responses
Source: Cell Res. 2025 Jan 6;35(2):97–116. doi: 10.1038/s41422-024-01060-w (PMC11770101; doi:10.1038/s41422-024-01060-w)
Supplement: Supplementary file 2 — Supplementary information, Fig. S2 [file 41422_2024_1060_MOESM2_ESM.pdf]

## Supplementary information, Fig. S2

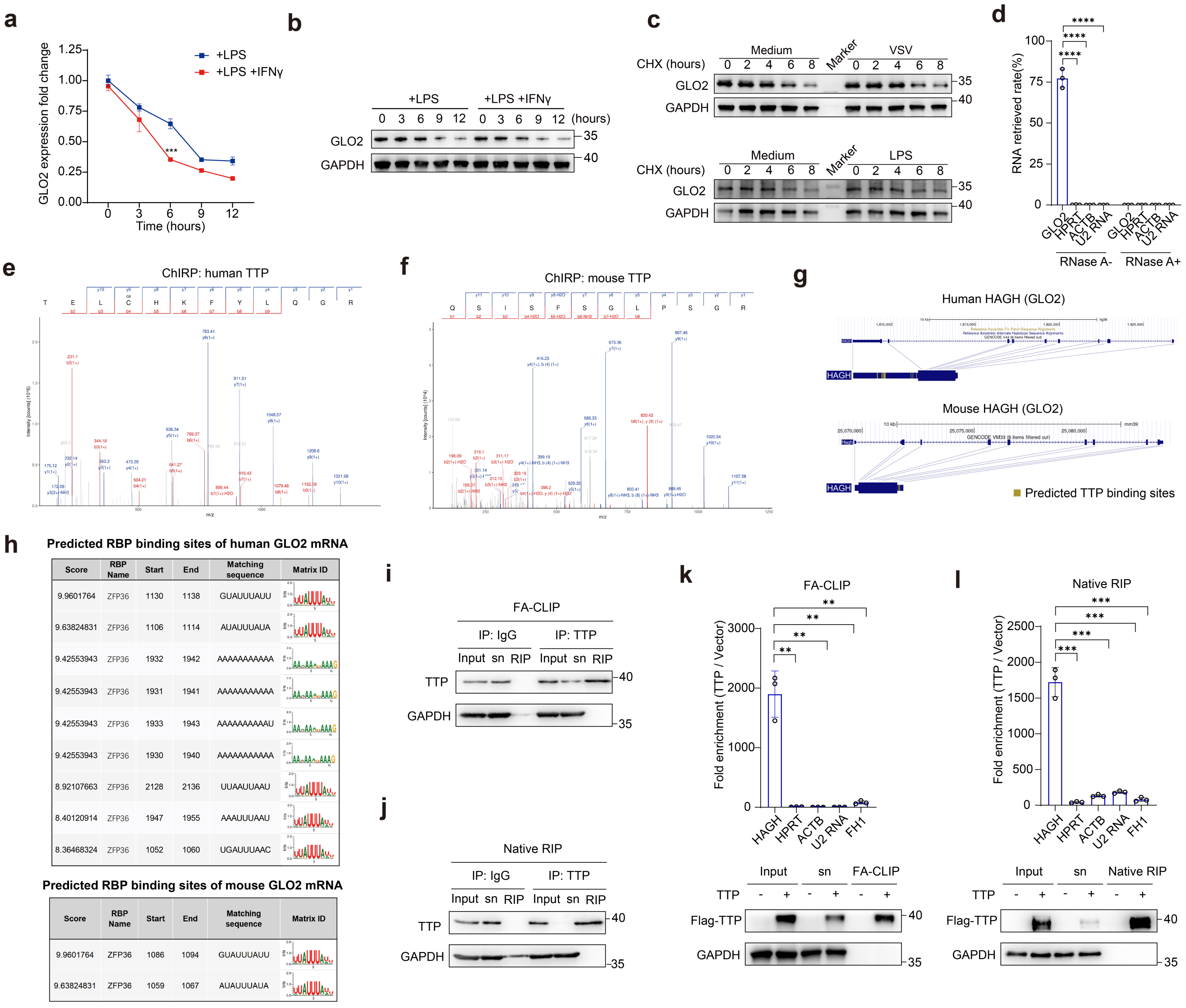


**Fig. S2 TTP binds to the ARE sites of GLO2 mRNA and mediates its decay. a, b,** Q-PCR and immunoblot analysis of GLO2 levels in PMs stimulated by LPS (100ng/mL) or LPS with IFNγ (50ng/mL). **c,** Immunoblot detection of degradation rates of GLO2 protein on VSV, LPS, or medium control treated BMDMs meanwhile adding CHX (100 μg/mL). **d,** Q-PCR analysis of GLO2 mRNA retrieving efficiency and probe specificity in the ChIRP assay. **e, f,** LC-MS/MS spectrum of mouse **(e)** and human TTP proteins **(f)** enriched from the ChIRP assay. **g, h,** TTP-binding sites in the human GLO2 mRNA as predicted by the RBPDB database. **i, j,** Immunoblot detection of TTP protein after FA-CLIP **(i)** and native-RIP **(j)** from BMDM cells. **k,** FA-CLIP analysis of the binding ability of TTP protein with indicated mRNAs upon Flag-TTP overexpression in HEK-

293T cells. **l,** native-RIP analysis of the binding ability of TTP protein with indicated mRNAs upon Flag-TTP overexpression in HEK-293T cells.
