# Supplementary material for: Nonenzymatic lysine d-lactylation induced by glyoxalase II substrate SLG dampens inflammatory immune responses
Source: Cell Res. 2025 Jan 6;35(2):97–116. doi: 10.1038/s41422-024-01060-w (PMC11770101; doi:10.1038/s41422-024-01060-w)
Supplement: Supplementary file 3 — Supplementary information, Fig. S3 [file 41422_2024_1060_MOESM3_ESM.pdf]

Supplementary information, Fig. S3

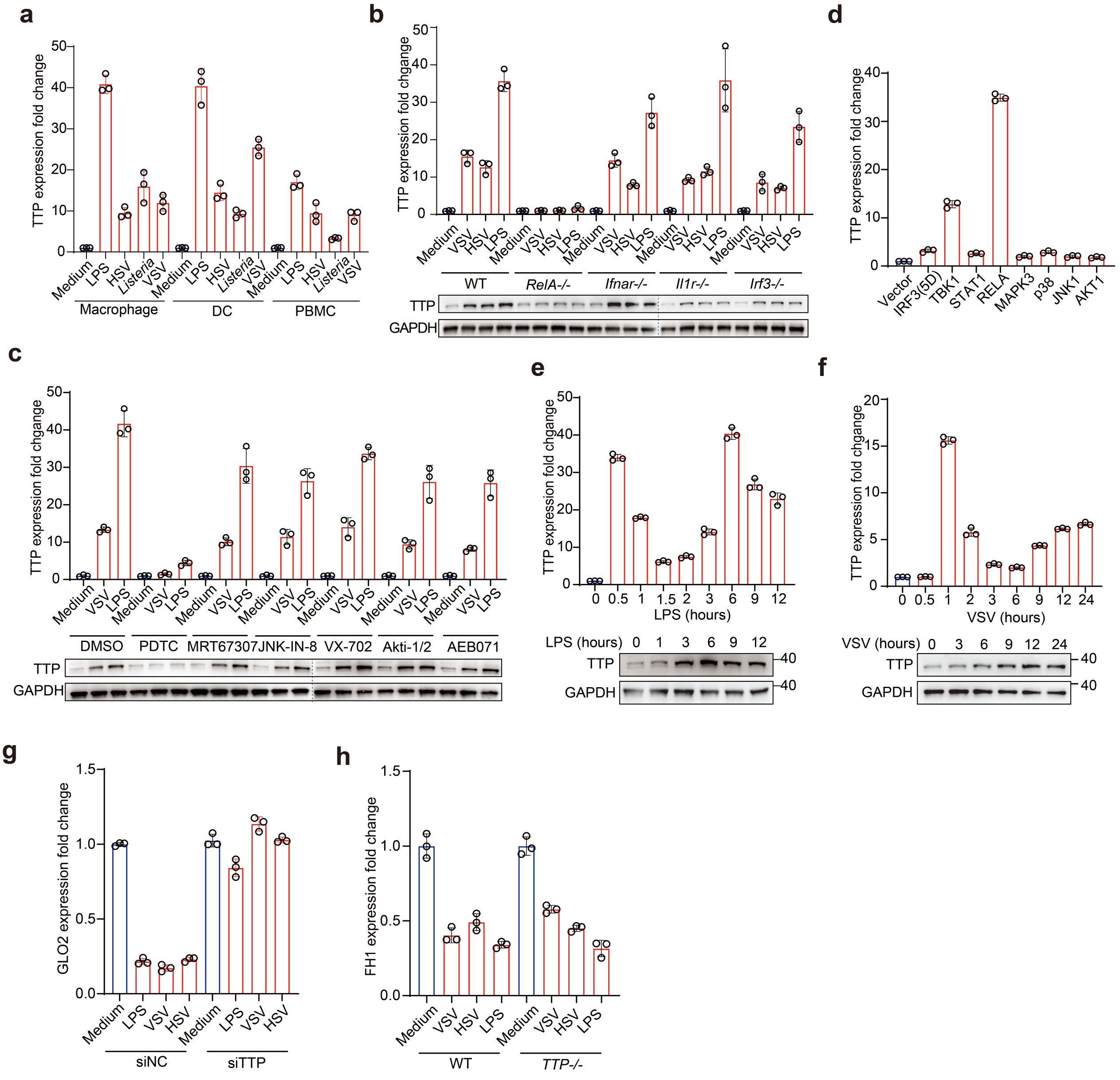

Fig. S3 TTP expression controls the level of GLO2 in immune cells. a, Q-PCR analysis of TTP expression in indicated cells stimulated as indicated. **b, c,** Q-PCR and immunoblot analysis of indicated gene expression in gene-knockout **(b)** or inhibitor-pretreated **(c)** BMDMs stimulated as indicated. **d,** Q-P0CR analysis of TTP expression in HEK-293T cells overexpressing indicated genes for 24 hours. **e, f,** Q-PCR and immunoblot analysis of TTP expression in BMDMs stimulated by LPS **(e)** or VSV **(f)**. **g,** Q-PCR analysis of GLO2 expression in control and TTP knockdown BMDMs

stimulated as indicated. **h,** Q-PCR analysis of FH1 expression in wild-type and TTP knockout BMDMs stimulated as indicated.
