# Supplementary material for: Nonenzymatic lysine d-lactylation induced by glyoxalase II substrate SLG dampens inflammatory immune responses
Source: Cell Res. 2025 Jan 6;35(2):97–116. doi: 10.1038/s41422-024-01060-w (PMC11770101; doi:10.1038/s41422-024-01060-w)
Supplement: Supplementary file 4 — Supplementary information, Fig. S4 [file 41422_2024_1060_MOESM4_ESM.pdf]

Supplementary information, Fig. S4

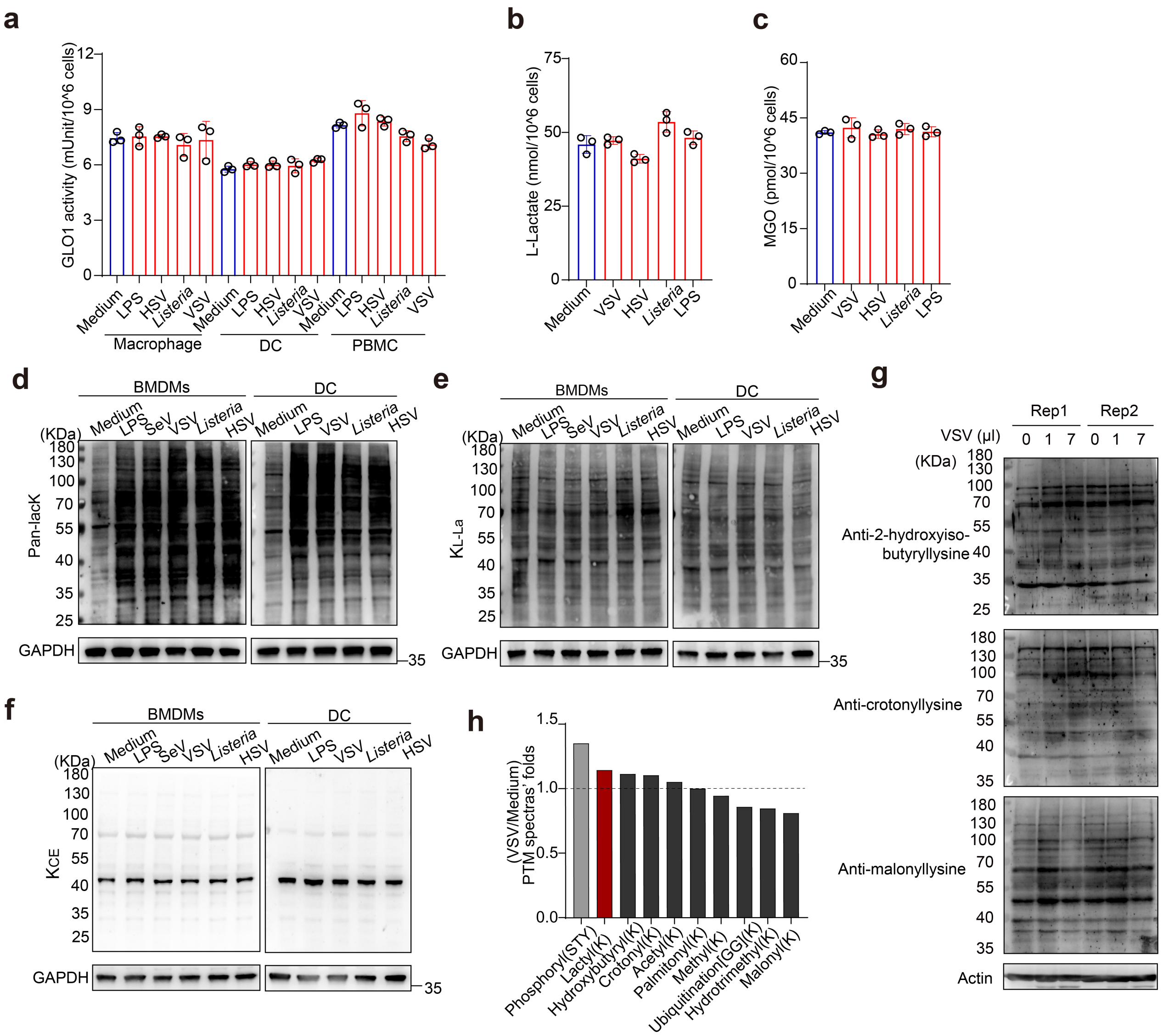

i

MS/MS spectrum database search analysis summary (Localization probability > 0.75)

Total spectrums	Matched spectrums	Peptides	Modified peptides	Identified proteins	Quantifiable proteins	Identified sites	Quantifiable sites
389893	67401	14414	2243	901	380	2255	884

Differentially modified sites (modified proteins) summary

Compare group	Regulated type	fold change >1.2	fold change >1.3	fold change >1.5	fold change >2
V/C	Up-regulated	378 (204)	378 (204)	376 (203)	323 (193)
	Down-regulated	0 (0)	0 (0)	0 (0)	0 (0)

Fig. S4 Immune activation induces SLG accumulation and increasing of *D*-lactylation. a, Enzymatic activity detection of GLO1 in innate immune cells stimulated as indicated. **b, c,** Relative quantification of *L*-lactate **(b)** and MGO **(c)** in BMDMs stimulated as indicated. **d, e, f,** Immunoblot of pan-LacK **(d)**, $K_{L\text{-}La}$ **(e)**, and K_{CE} **(f)**

levels in innate immune cells stimulated as indicated. **g,** Immunoblot detection of indicated acylation levels in mouse peritoneal macrophages with stimulation of VSV. **h,** Analysis of indicated lysine acylation modification in proteome data from mouse peritoneal macrophages stimulated by VSV versus medium-alone control. Different types of acylation sites were analyzed by pFind 3.1 software. **i,** MS/MS spectrum database search analysis summary of antibody-enriched lacK peptides identified by LC-MS/MS in peritoneal macrophages activated by VSV (V) versus medium-alone control (C).
