# Supplementary material for: Nonenzymatic lysine d-lactylation induced by glyoxalase II substrate SLG dampens inflammatory immune responses
Source: Cell Res. 2025 Jan 6;35(2):97–116. doi: 10.1038/s41422-024-01060-w (PMC11770101; doi:10.1038/s41422-024-01060-w)
Supplement: Supplementary file 5 — Supplementary information, Fig. S5 [file 41422_2024_1060_MOESM5_ESM.pdf]

Supplementary information, Fig. S5

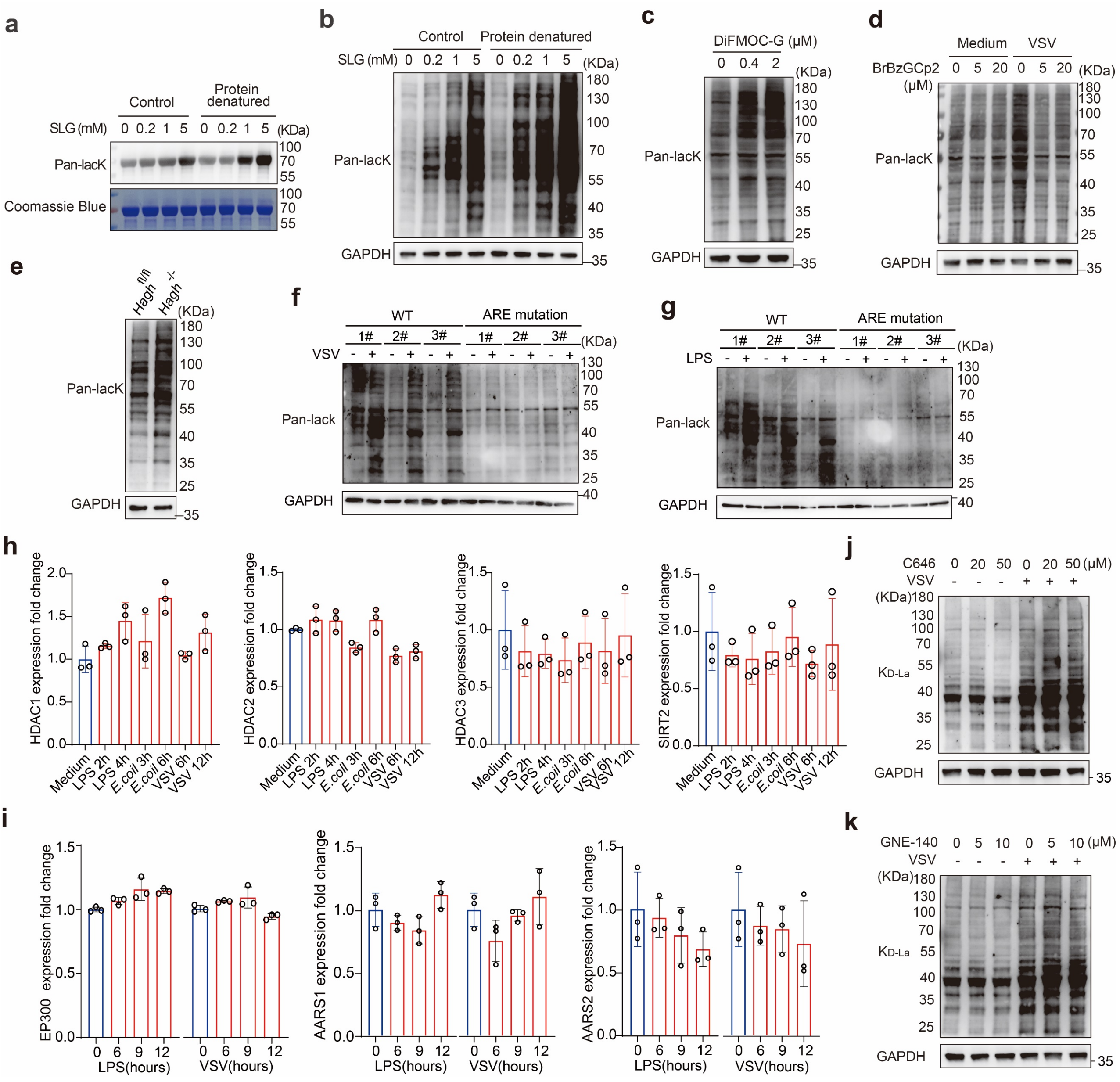

Fig. S5 SLG mediates non-enzymatic *D*-lactylation in activated immune cells. a, b, Immunoblot of pan-lacK levels in BSA **(a)** or macrophage lysate **(b)** denatured by boiling or not, then co-incubated with indicated concentration of SLG for 4 hours at 37°C. **c,** Immunoblot of pan-lacK levels in BMDMs pretreated with indicated concentrations of DiFMOC-G for 24 hours. **d,** Immunoblot of pan-lacK levels in BMDMs pretreated with indicated concentrations of BrBzGCP2 then stimulated by VSV or not. **e,** Immunoblot detection of pan-lacK levels in *Hagh*$^{+/+}$ and *Hagh*$^{-/-}$ BMDMs. **f, g,** Immunoblot of pan-lacK levels in wild-type or ARE mutation RAW

264.7 cell lines infected by VSV **(f)** or LPS **(g)** for 12 hours**. h, i,** Q-PCR analysis of gene expression of the reported lactylation “erasers” **(h)** or “writers” **(i)** in BMDMs stimulated as indicated. **j, k,** Immunoblot detection of $K_{D\text{-}La}$ levels in BMDMs treated with indicated concentrations of P300 inhibitor C646 **(j)** or LDHA inhibitor GNE-140 **(k)** then infected by VSV (12h).
