# Supplementary material for: Nonenzymatic lysine d-lactylation induced by glyoxalase II substrate SLG dampens inflammatory immune responses
Source: Cell Res. 2025 Jan 6;35(2):97–116. doi: 10.1038/s41422-024-01060-w (PMC11770101; doi:10.1038/s41422-024-01060-w)
Supplement: Supplementary file 6 — Supplementary information, Fig. S6 [file 41422_2024_1060_MOESM6_ESM.pdf]

Supplementary information, Fig. S6

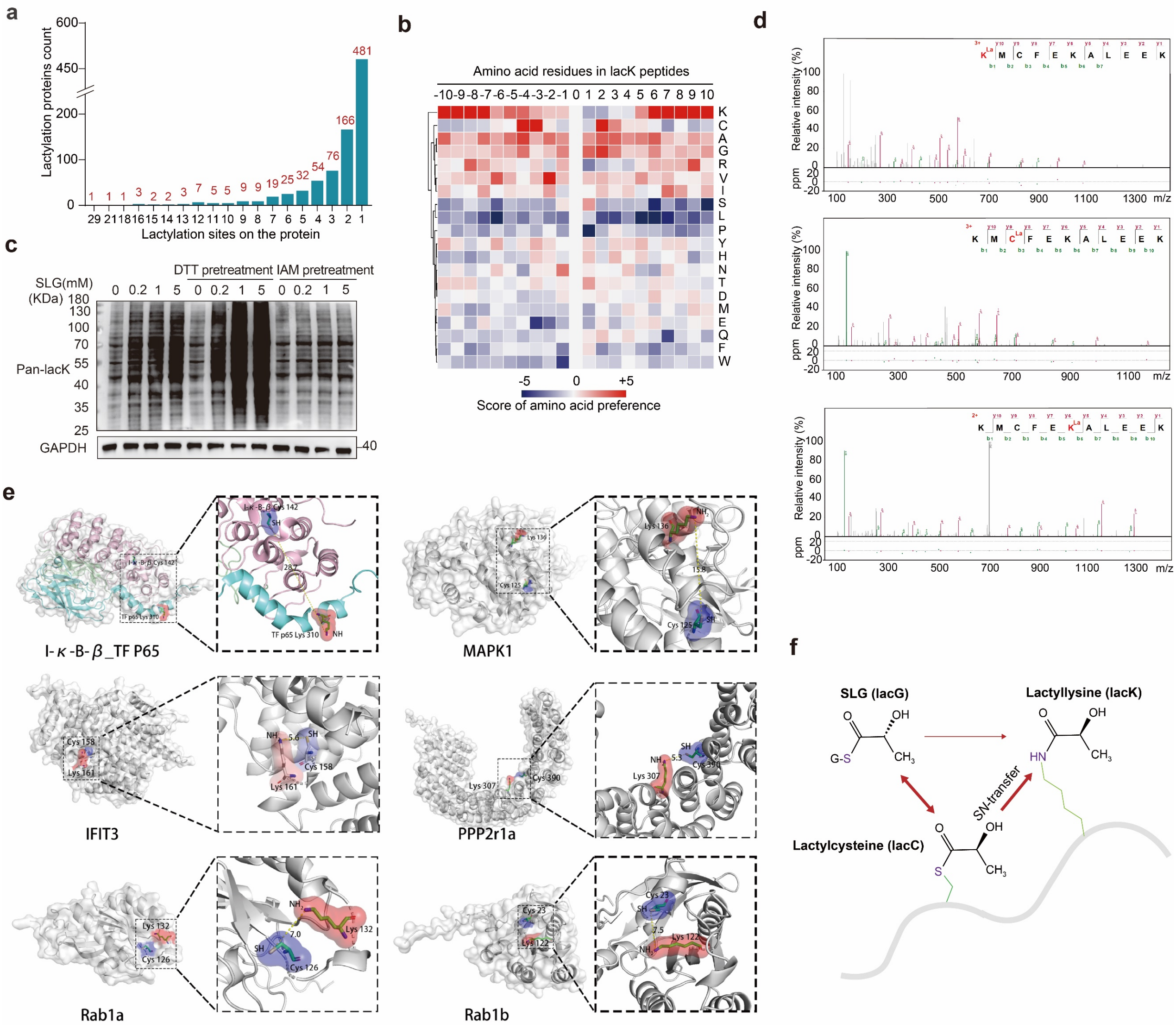

Fig. S6 SLG-induced lacK modification is facilitated by its nearby cysteine residue. a, Counting of proteins with different number lacK sites identified in macrophages by antibody-enriched lactylome profiling. **b,** Heatmap of amino acid preference on lacK peptides identified in macrophages. **c,** Immunoblot of pan-lacK level in BMDM lysates pretreated with DTT (5mM) or (10mM), then co-incubated with the indicated concentration of SLG**. d,** MS/MS spectra of lacC or lacK sites on wild-type or mutated IFIT3$_{156\text{-}166}$ peptides pretreated with IAM (10mM) or not followed by SLG (1mM) coincubation. **e,** Structure of indicated proteins from AlphaFold database and the distance from lactylated lysine residues to its nearest cysteine residue. **f,** *SN*-transfer of

lactyl moiety from adjacent cysteine to lysine residue facilitates SLG-induced non-enzymatic lysine lactylation.
