# Supplementary material for: Nonenzymatic lysine d-lactylation induced by glyoxalase II substrate SLG dampens inflammatory immune responses
Source: Cell Res. 2025 Jan 6;35(2):97–116. doi: 10.1038/s41422-024-01060-w (PMC11770101; doi:10.1038/s41422-024-01060-w)
Supplement: Supplementary file 7 — Supplementary information, Fig. S7 [file 41422_2024_1060_MOESM7_ESM.pdf]

Supplementary information, Fig. S7

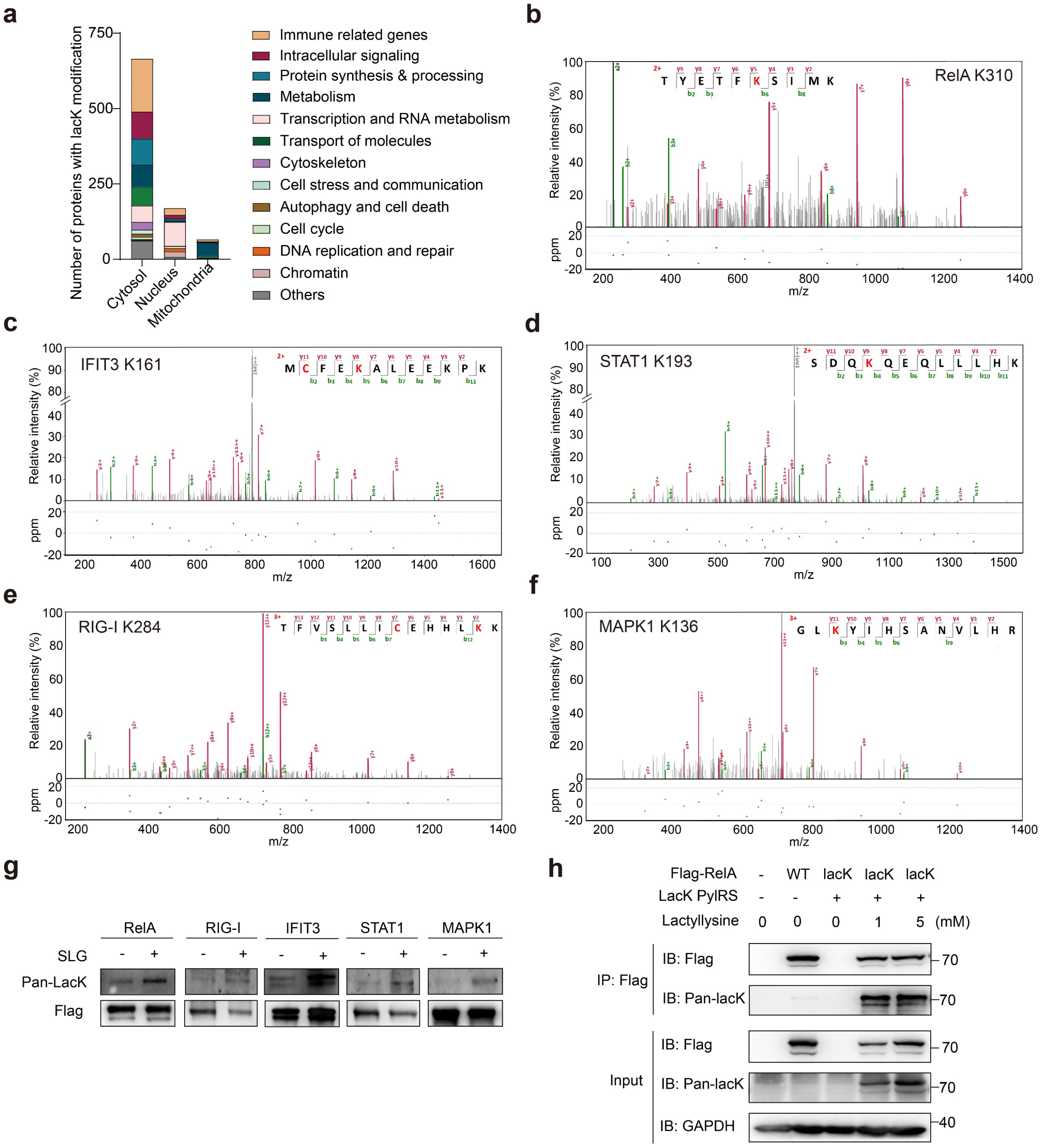

Fig. S7 LacK modification on immune-related proteins attenuates the downstream signaling. a, Subcellular localization and functional statistics of 901 proteins with lacK modification identified in viral-stimulated macrophages. **b-f,** MS/MS spectra of indicated lactylation peptides analyzed by pFind 3.1 software from the proteome of mouse macrophages stimulated by VSV. **g,** Immunoblot detection of lacK modification

on indicated proteins after co-incubation with or without SLG (1mM). **h,** Immunoblot detection of site-specific lactylated RelA proteins (lacK310) expressed by orthogonal lacyl-tRNA synthetase (LacK PylRS) and tRNA pairs in HEK-293T cells.
