# Supplementary material for: Nonenzymatic lysine d-lactylation induced by glyoxalase II substrate SLG dampens inflammatory immune responses
Source: Cell Res. 2025 Jan 6;35(2):97–116. doi: 10.1038/s41422-024-01060-w (PMC11770101; doi:10.1038/s41422-024-01060-w)
Supplement: Supplementary file 8 — Supplementary information, Fig. S8 [file 41422_2024_1060_MOESM8_ESM.pdf]

Supplementary information, Fig. S8

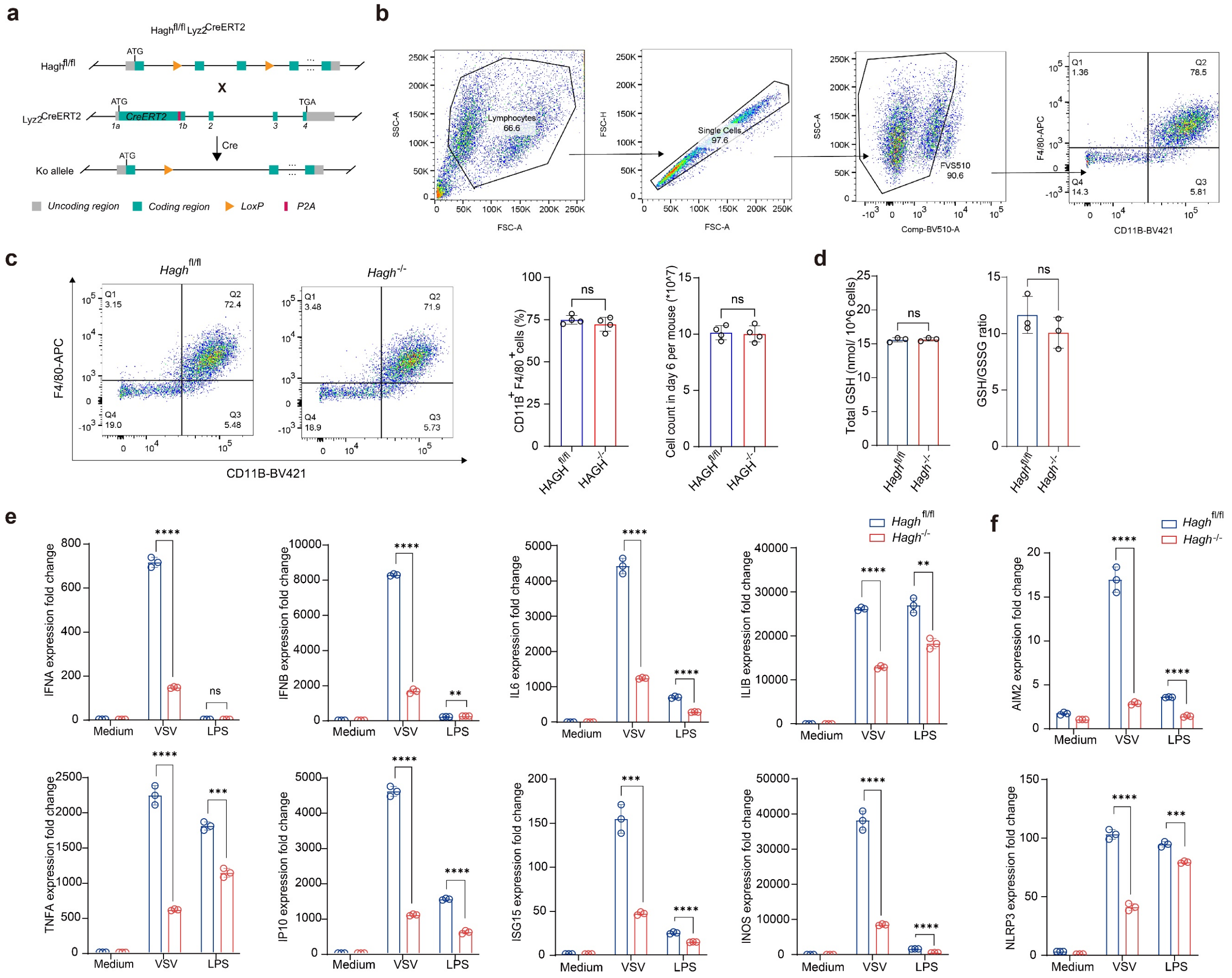

Fig. S8 Knockout of GLO2 inhibit inflammation levels by attenuating interferon and inflammatory cytokine production. a, Genetic construction and hybridization of *Hagh*$^{fl/fl}$*Lyz2*^{creERT2} mice. **b, c,** Gating strategy **(b)** and detection **(c)** of CD11B^{+}F4/80^{+} BMDMs from *Hagh*$^{fl/fl}$ or *Hagh*$^{fl/fl}$*Lyz2*^{creERT2} mice on the induce and culture day 7. **d,** Detection of GSH and GSSG levels in *Hagh*$^{fl/fl}$ and *Hagh*$^{fl/fl}$*Lyz2*^{creERT2} BMDMs**. e, f,** Q-PCR analysis of indicated mRNA levels in BMDMs from *Hagh*$^{fl/fl}$ and *Hagh*$^{fl/fl}$*Lyz2*^{creERT2} mice and after stimulation as indicated.
