# Supplementary material for: Nonenzymatic lysine d-lactylation induced by glyoxalase II substrate SLG dampens inflammatory immune responses
Source: Cell Res. 2025 Jan 6;35(2):97–116. doi: 10.1038/s41422-024-01060-w (PMC11770101; doi:10.1038/s41422-024-01060-w)
Supplement: Supplementary file 9 — Supplementary information, Fig. S9 [file 41422_2024_1060_MOESM9_ESM.pdf]

Supplementary information, Fig. S9

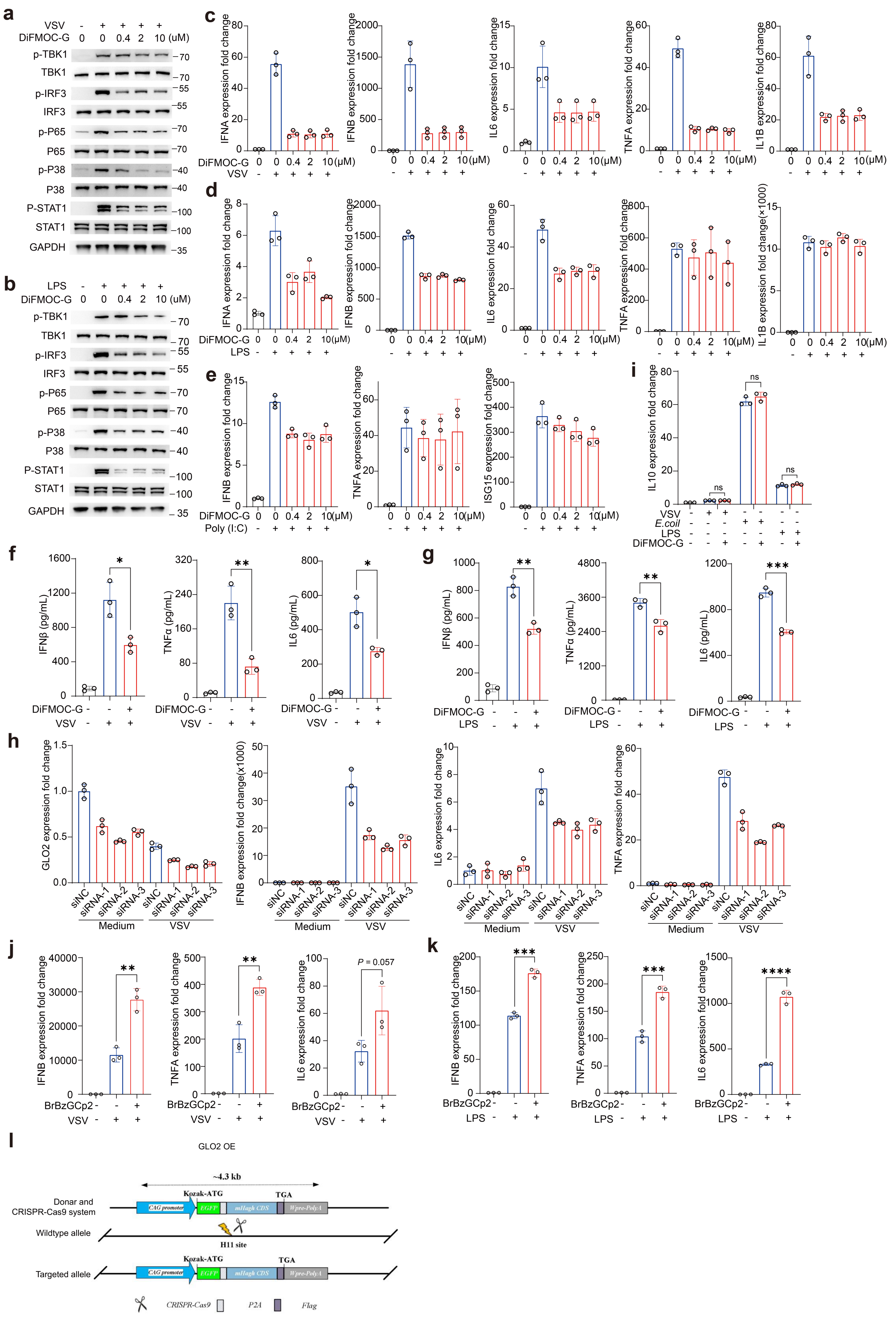

Fig. S9 Pharmacologic inhibition or knockdown of GLO2 decreases inflammatory signaling and cytokine production in vitro. a, b, Immunoblot detection of indicated proteins and its phosphorylation in BMDMs pretreated with indicated concentrations of DiFMOC-G for 12 hours then stimulated by VSV (8h) **(a)** or LPS(2h) **(b)**. **c, d, e,** Q-PCR analysis of indicated mRNAs in mouse BMDMs stimulated by VSV (6h, MOI = 1) **(c)**, LPS (2h, 400ng/mL) **(d)**, or Poly (I:C) (4h, 100μg/mL) **(e)** pre-treated with indicated concentrations of DiFMOC-G for 12 hours. **f, g,** ELISA detection of indicated cytokines secreted into supernatants by mouse BMDMs stimulated with VSV (8h, MOI = 0.1) **(f)** or LPS (6h, 400ng/mL) **(g)** with 12 hours pre-treatment of DiFMOC-G (0.4μM). **h,** Q-PCR analysis to quantify indicated mRNA expression in BMDMs stimulated by VSV (6h, MOI = 1) pre-treated with RNAi by transfection of different siRNAs targeting GLO2 mRNA for 24h. **i,** Q-PCR analysis of IL10 mRNA expression in BMDMs stimulated by VSV (9h, MOI = 0.1), LPS (2h, 400ng/mL), or *E. Coli* (9h, MOI = 10) with pre-treatment of DiFMOC-G (0.4μM) for 12 hours. **j, k,** Q-PCR analysis of indicated mRNA's expression in BMDMs stimulated by VSV (6h, MOI = 1) **(j)** or LPS (2h, 400ng/mL) **(k)** with pre-treatment of 5mM GLO1 inhibitor BrBzGCP2 for 12 hours. **l,** Genetic construction of GLO2 OE mice.
