# Supplementary material for: Nonenzymatic lysine d-lactylation induced by glyoxalase II substrate SLG dampens inflammatory immune responses
Source: Cell Res. 2025 Jan 6;35(2):97–116. doi: 10.1038/s41422-024-01060-w (PMC11770101; doi:10.1038/s41422-024-01060-w)
Supplement: Supplementary file 10 — Supplementary information, Fig. S10 [file 41422_2024_1060_MOESM10_ESM.pdf]

Supplementary information, Fig. S10

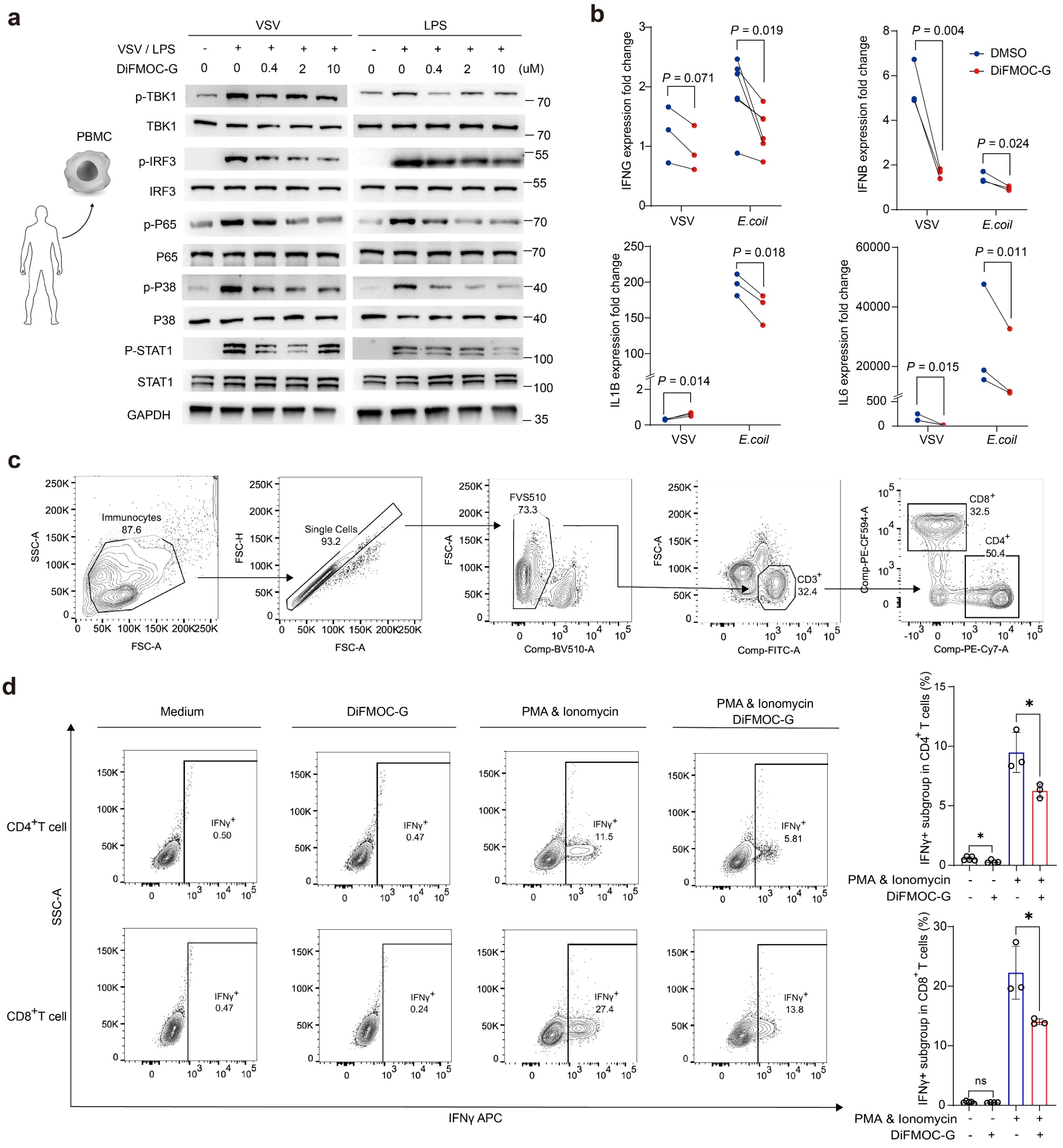

Fig. S10 Inhibition of GLO2 decreases interferon and inflammatory cytokine production in human immunocytes. a, Immunoblot detection of indicated proteins and its phosphorylation in human PBMC pretreated with indicated concentrations of DiFMOC-G for 12 hours then stimulated by VSV (8h) or LPS(2h). **b,** Q-PCR analysis of indicated mRNAs in human PBMCs stimulated with VSV (6h, MOI = 1) or *E. coli* (6h, MOI = 10) with 12 hours pre-treatment of DiFMOC-G for indicated concentrations.

c, d, Gating strategy **(c)** and flow cytometry detection **(d)** of IFNγ^+ secretion in CD4$^+$ and CD8$^+$ T cells of human PBMCs stimulated with PMA/ionomycin for 5 hours with 12 hours pre-treatment of DiFMOC-G (0.4μM).
