# Supplementary material for: Nonenzymatic lysine d-lactylation induced by glyoxalase II substrate SLG dampens inflammatory immune responses
Source: Cell Res. 2025 Jan 6;35(2):97–116. doi: 10.1038/s41422-024-01060-w (PMC11770101; doi:10.1038/s41422-024-01060-w)
Supplement: Supplementary file 11 — Supplementary information, Fig. S11 [file 41422_2024_1060_MOESM11_ESM.pdf]

Supplementary information, Fig. S11

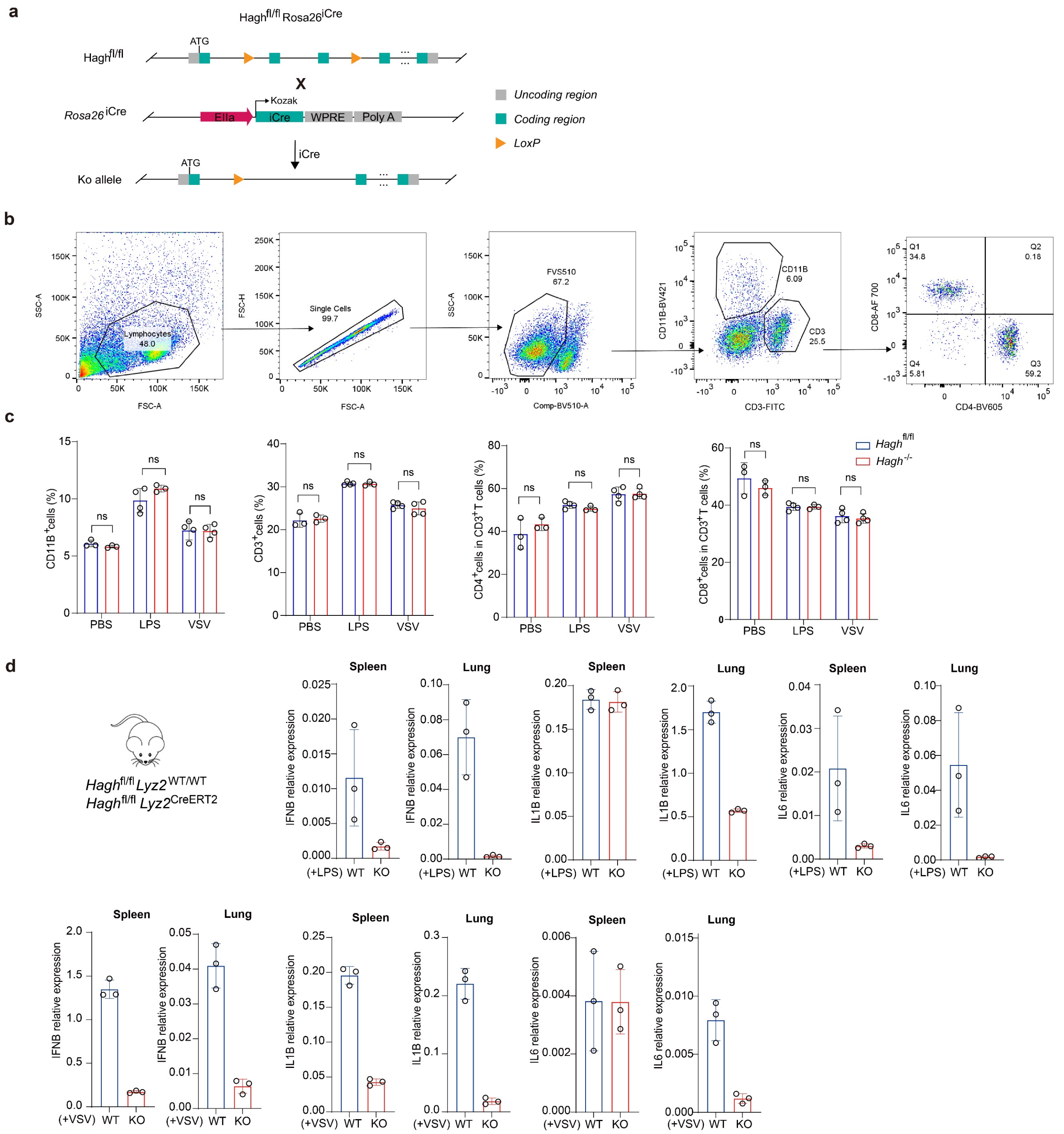

Fig. S11 Knockout of GLO2 decreases interferon and inflammatory cytokine production in vivo. a, Genetic construction and hybridization of *Hagh*$^{fl/fl}$*Rosa26*^{icre} mice**. b, c,** Gating strategy **(b)** and detection of indicated immune cell subgroups from the spleen of *Hagh*$^{fl/fl}$ or *Hagh*$^{fl/fl}$*Rosa26*^{icre} mice **(c). d,** Q-PCR analysis of indicated mRNAs on indicated tissues from control or GLO2 knockout mice i.p. injected with VSV (1 × 10^{7}pfu/g) or LPS (80μg/g) for 12 hours.
