# Supplementary material for: Nonenzymatic lysine d-lactylation induced by glyoxalase II substrate SLG dampens inflammatory immune responses
Source: Cell Res. 2025 Jan 6;35(2):97–116. doi: 10.1038/s41422-024-01060-w (PMC11770101; doi:10.1038/s41422-024-01060-w)
Supplement: Supplementary file 12 — Supplementary information, Fig. S12 [file 41422_2024_1060_MOESM12_ESM.pdf]

Supplementary information, Fig. S12

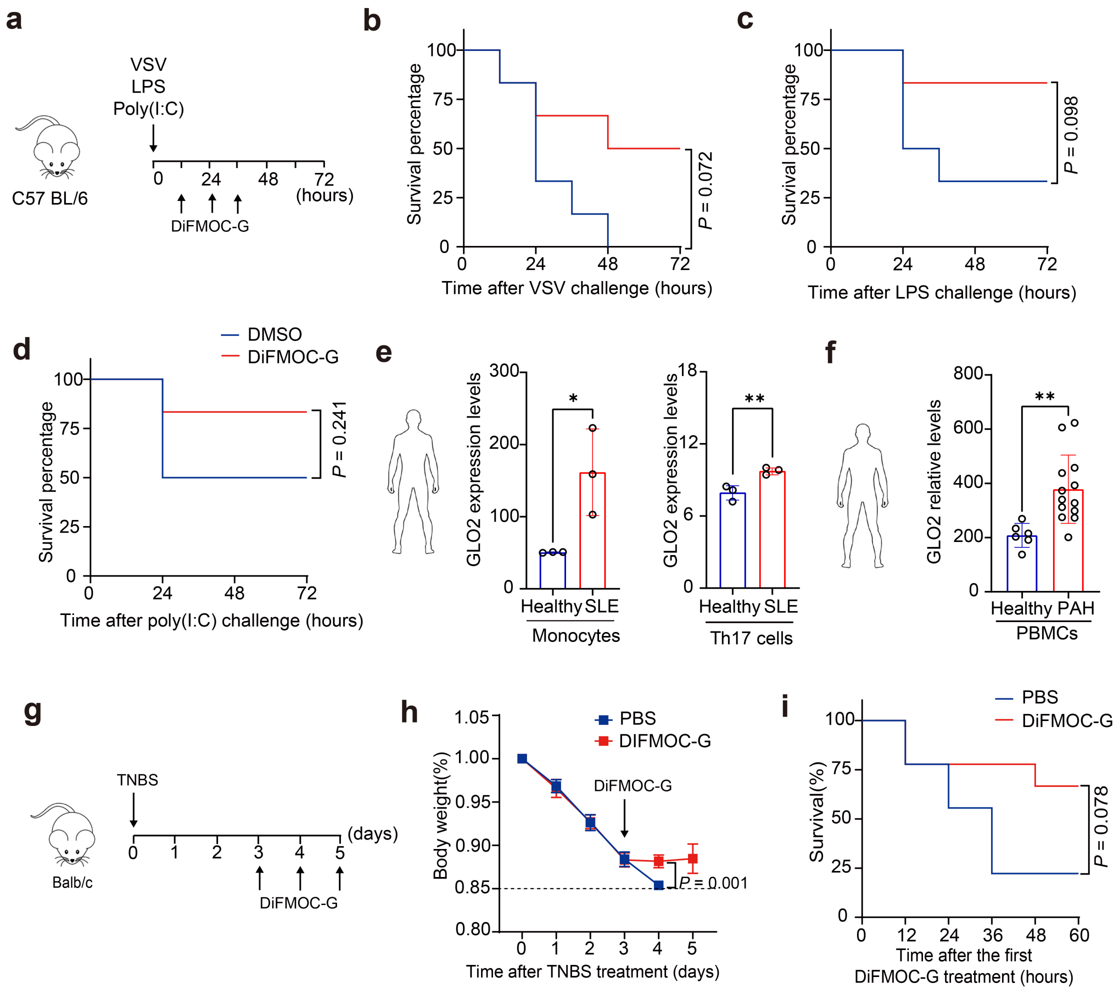

Fig. S12 GLO2 as a potential target for treating inflammatory diseases. a, Experiment design of anti-inflammation function assay of DiFMOC-G on acute inflammation and cytokine storm mouse model. **b, c, d,** Survival of mice pretreated with VSV **(b)**, LPS **(c)**, or intravenous (i.v.) injected with Poly (I:C) **(d)**, and then i.p. injected with DiFMOC-G (800mg/kg). **e,** GLO2 levels in monocytes and Th17 cells from healthy donors and SLE patients (GSE218492, GSE212663). **f,** GLO2 levels in PBMCs from healthy donors and inflammation-related severe pulmonary hypertension (PAH) patients (GSE703). **g,** Experimental design of anti-inflammation function assay of DiFMOC-G for the TNBS-induced colitis model. **h,** Body weight of TNBS-induced colitis mice i.p. treated with or without DiFMOC-G. **i,** Survival of colitis mice i.p. treated with or without DiFMOC-G.
