# Supplementary material for: Nonenzymatic lysine d-lactylation induced by glyoxalase II substrate SLG dampens inflammatory immune responses
Source: Cell Res. 2025 Jan 6;35(2):97–116. doi: 10.1038/s41422-024-01060-w (PMC11770101; doi:10.1038/s41422-024-01060-w)
Supplement: Supplementary file 14 — Supplementary information, Table S2 [file 41422_2024_1060_MOESM14_ESM.pdf]

Table. S2. Sequence motif of lactylation analyzed by identified lactylation peptides.

Motif Logo	Motif	Motif Score	Foreground		Background		Fold Increase
			Matches	Size	Matches	Size	
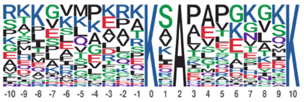	xxxxxxxxxx_K_xAxxxxxxxK	20.08	41	2254	2335	518092	4.0
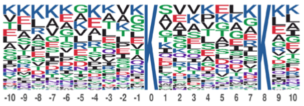	xxxxxxxxxx_K_xxxxxxxKxx	11.11	254	2213	38313	515757	1.5
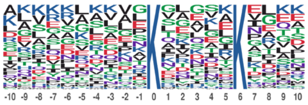	xxxxxxxxxx_K_xxxxxKxxxx	10.17	223	1959	34886	477444	1.6
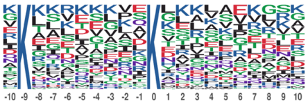	xKxxxxxxxx_K_xxxxxxxxxx	8.64	193	1736	31840	442558	1.5
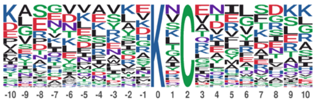	xxxxxxxxxx_K_xCxxxxxxxx	7.56	69	1543	8859	410718	2.1
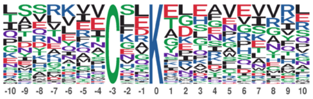	xxxxxxxCxx_K_xxxxxxxxxx	6.66	59	1474	7708	401859	2.1
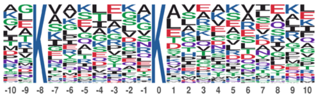	xxKxxxxxxx_K_xxxxxxxxxx	6.41	150	1415	27562	394151	1.5
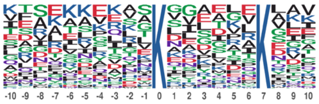	xxxxxxxxxx_K_xxxxxxKxxx	6.61	137	1265	25432	366589	1.6
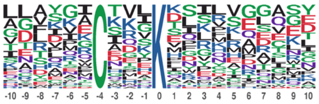	xxxxxxCxxx_K_xxxxxxxxxx	6.59	49	1128	6582	341157	2.3
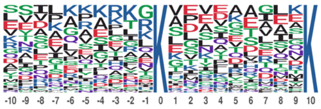	xxxxxxxxxx_K_xxxxxxxxxK	6.89	112	1079	20815	334575	1.7
